# Deletion of genomic islands in the *Pseudomonas putida* KT2440 genome can create an optimal chassis for synthetic biology applications

**DOI:** 10.1186/s12934-020-01329-w

**Published:** 2020-03-18

**Authors:** Peixin Liang, Yiting Zhang, Bo Xu, Yuxin Zhao, Xiangsheng Liu, Weixia Gao, Ting Ma, Chao Yang, Shufang Wang, Ruihua Liu

**Affiliations:** 1 Key Laboratory of Molecular Microbiology and Technology for Ministry of Education, Nankai University, Tianjin, 300071 China; 2 State Key Laboratory of Medicinal Chemical Biology, Nankai University, Tianjin, 300071 China

**Keywords:** Genomic islands, Chassis, Synthetic biology, *Pseudomonas putida* KT2440

## Abstract

**Background:**

Genome streamlining is a feasible strategy for constructing an optimum microbial chassis for synthetic biology applications. Genomic islands (GIs) are usually regarded as foreign DNA sequences, which can be obtained by horizontal gene transfer among microorganisms. A model strain *Pseudomonas putida* KT2440 has broad applications in biocatalysis, biotransformation and biodegradation.

**Results:**

In this study, the identified GIs in *P. putida* KT2440 accounting for 4.12% of the total genome size were deleted to generate a series of genome-reduced strains. The mutant KTU-U13 with the largest deletion was advantageous over the original strain KTU in several physiological characteristics evaluated. The mutant KTU-U13 showed high plasmid transformation efficiency and heterologous protein expression capacity compared with the original strain KTU. The metabolic phenotype analysis showed that the types of carbon sources utilized by the mutant KTU-U13 and the utilization capabilities for certain carbon sources were increased greatly. The polyhydroxyalkanoate (PHA) yield and cell dry weight of the mutant KTU-U13 were improved significantly compared with the original strain KTU. The chromosomal integration efficiencies for the γ-hexachlorocyclohexane (γ-HCH) and 1,2,3-trichloropropane (TCP) biodegradation pathways were improved greatly when using the mutant KTU-U13 as the recipient cell and enhanced degradation of γ-HCH and TCP by the mutant KTU-U13 was also observed. The mutant KTU-U13 was able to stably express a plasmid-borne zeaxanthin biosynthetic pathway, suggesting the excellent genetic stability of the mutant.

**Conclusions:**

These desirable traits make the GIs-deleted mutant KTU-U13 an optimum chassis for synthetic biology applications. The present study suggests that the systematic deletion of GIs in bacteria may be a useful approach for generating an optimal chassis for the construction of microbial cell factories.

## Background

Studies on genome sequencing and functional genes of many model organisms have found that microorganisms absorb exogenous DNA sequences and retain them in their own genome through gene horizontal transfer and other means in the long evolutionary process, resulting in increased genome capacity. Moderate genome reduction can optimize cellular metabolic pathways, improve cellular utilization efficiency of substrates and energy, enhance recombinant protein productivity, and improve the predictability and controllability of cellular physiological performance [1–3].

Genome streamlining is an important strategy for constructing synthetic chassis. Genome-streamlined strains provide a basic platform for the introduction of functional modules. Choosing an optimum chassis can make the modules work efficiently, resulting in an ideal cell factory. The minimum genome factory has broad application prospects in the fields of metabolite production, environmental waste degradation, cytotoxin detection and biofuel production [4–6].

In previous studies, two genome-reduced strains EM329 and EM383 were constructed by deleting 1.1 and 4.3% of the *P. putida* KT2440 genome, respectively. The deleted genomic regions include the flagellar operon, prophages 1, 2, 3 and 4, deoxyribonucleases I and II, transposases Tn7 and Tn4652, recombinase A, and type I restriction-modification system. Compared with the wild-type strain KT2440, the genome-reduced strain EM383 showed many excellent properties such as faster growth, increased biomass, higher cell viability, increased plasmid stability, more efficient energy metabolism, and higher expression level of heterologous genes [7, 8]. In another study, a series of *Bacillus subtilis* 168 mutant strains with genome reductions ranging in size from 581.9 to 814.4 kb were constructed by deleting non-essential regions in the chromosome of *B. subtilis* 168. Furthermore, an efficient guanosine or thymidine producer was developed with the genome-reduced *B*. *subtilis* strains by introducing specific gene modifications related to guanosine and thymidine accumulation [9]. In a recent study, genome-reduced strains of *Lactococcus lactis* NZ9000 were constructed by deleting four large nonessential DNA regions, up to 2.83% of the *L. lactis* genome. *L. lactis* 9 k-4 with the largest deletion not only outcompeted the parental strain in several physiological traits assessed, but it also exhibited good properties as platform organism for heterologous protein production [10].

The genomic islands (GIs) are large DNA segments, generally between 10 and 200 kb in length, with special structure and function. GIs can always be horizontal transferable, and host bacteria can acquire GIs by conjugation, transduction and transformation [11, 12]. The common features of GIs include: (1) integration hotspots for GIs are usually adjacent to RNA genes on chromosome; (2) GIs are flanked by direct repeats that are possibly related to the horizontal transfer of GIs; (3) horizontal transfer related genes, such as transposases, integrases, and recombinases, are often found at the junction of GIs and core genome; (4) there are significant differences in G+C % content, oligonucleotide distribution and codon usage bias between GIs and core genome [13]. GIs have a variety of biological functions, such as antibiotic resistance, pathogenicity, xenobiotic degradation and heavy metal resistance. Currently, two main bioinformatic approaches have been proposed for identifying GIs, including sequence composition-based and comparative genomics-based methods [12].

*Pseudomonas putida* KT2440 has a genome size of 6,181,863 bp (GenBank no. AE015451) with an average G+C content of 61.6% [14]. Analysis of the *P. putida* KT2440 genome with the sequence composition-based approach showed that 105 DNA regions larger than 4 kb with a typical GIs sequence features are interspersed in the genome of this microorganism [15]. Furthermore, 61 putative GIs in the KT2440 genome were identified using sequence composition-based and comparative genomics-based methods [16]. The soil bacterium *P. putida* KT2440 is not only certified as GRAS (generally recognized as safe), but it also possesses diverse transport and metabolic systems, enabling it to function as a chassis in biocatalysis, heterologous production, biodegradation and bioplastics production [17, 18]. Moreover, the scarless genome editing methods have been well established in *P. putida* KT2440 [19–21], which have paved the way for genome streamlining in *P. putida* KT2440.

In this study, the GIs in *P. putida* KT2440 were identified and deleted using a marker-less large DNA fragment deletion method. The mutants were compared with the wild-type strain KT2440 in several physiological traits and evaluated as chassis cells for the production of endogenous products as well as the expression of heterologous metabolic pathways.

## Results

### Construction of GIs-deleted *P. putida* KT2440 mutants

**Fig. 1:**
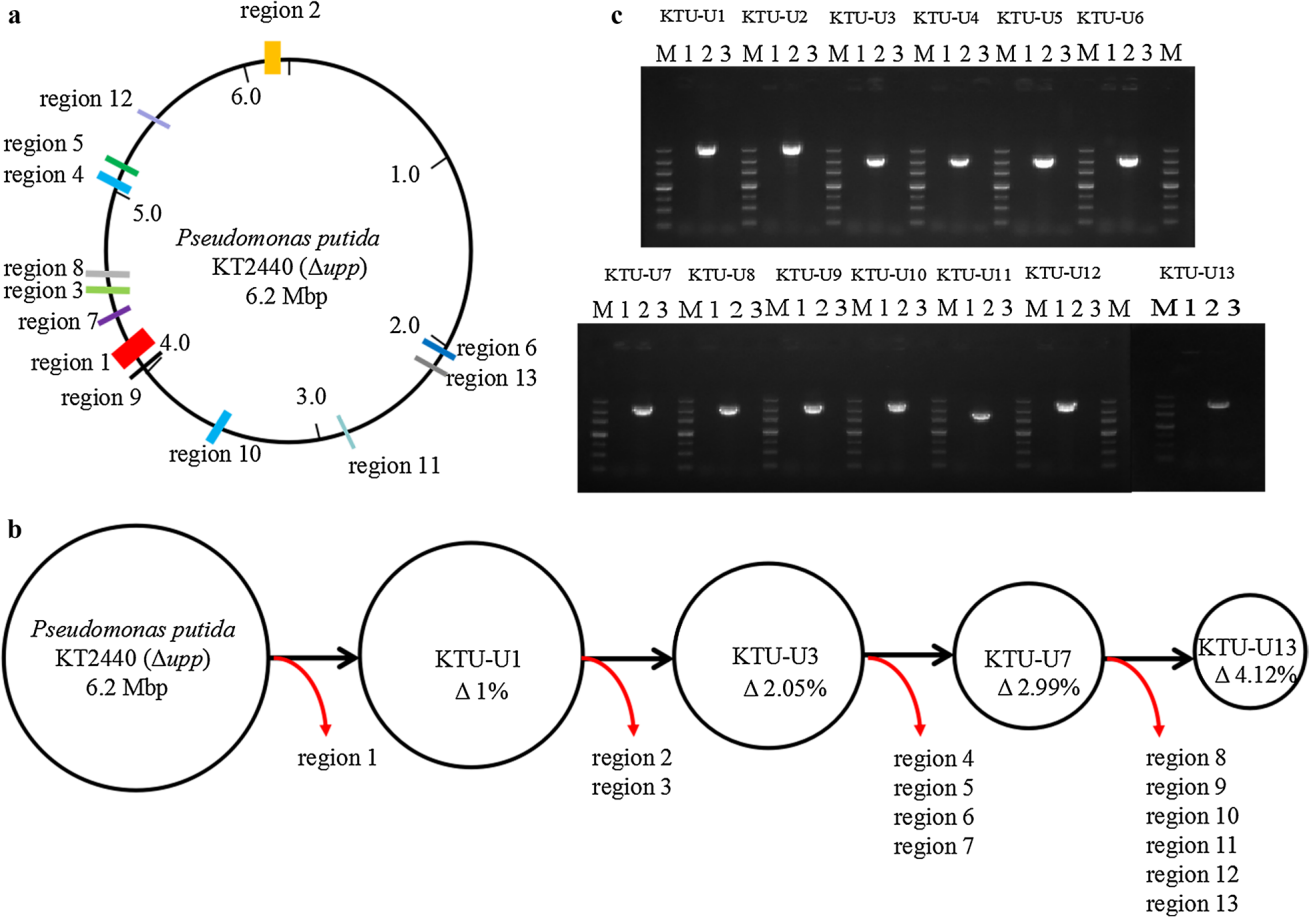
The construction of the GIs-deleted mutants of *P. putida* KT2440. **a** The physical localization of the deleted regions on the chromosome of *P. putida* KT2440. **b** A pipeline for constructing the GIs-deleted mutants of *P. putida* KT2440. **c** Confirmation of the deleted genomic regions in the *P. putida* mutants by PCR. Lanes: M, DNA marker; 1, the use of the genomic DNA of the control strain *P. putida* KTU as the template; 2, the use of the genomic DNA of the *P. putida* mutants as the template; 3, the use of ddH_2_O as the template

We initially searched the GIs in the *P. putida* KT2440 genome as the deletion targets for constructing the GIs-deleted mutants. Here, 43 GIs were predicted by using a GC-Profile from the Tianjin University Bioinformatics Center (TUBIC) (http://tubic.tju.edu.cn), and the GIs with a 5% difference from the average GC content of the KT2440 genome were selected as the deletion targets. The localization of the deleted regions in the chromosomal coordinates of *P. putida* KT2440 and the genomic manipulations in each mutant are summarized in Fig. 1a, b, respectively.

The schematic diagram for knockout of GIs in the *P. putida* genome is shown in Additional file 1: Fig. S1. Overall, the deleted genomic regions were accumulated to a total size of ~ 254.52 kb, accounting for ~ 4.12% of the KT2440 genome (Additional file 1: Tables S1 and S2). The successful deletion of the target genomic regions was tentatively verified by PCR detection using the specific primers (Fig. 1c). Furthermore, DNA sequencing of the amplified fragments indicated that the deleted genomic regions were in agreement with the expected results.

### Growth properties of the *P. putida* KT2440 mutants

**Fig. 2:**
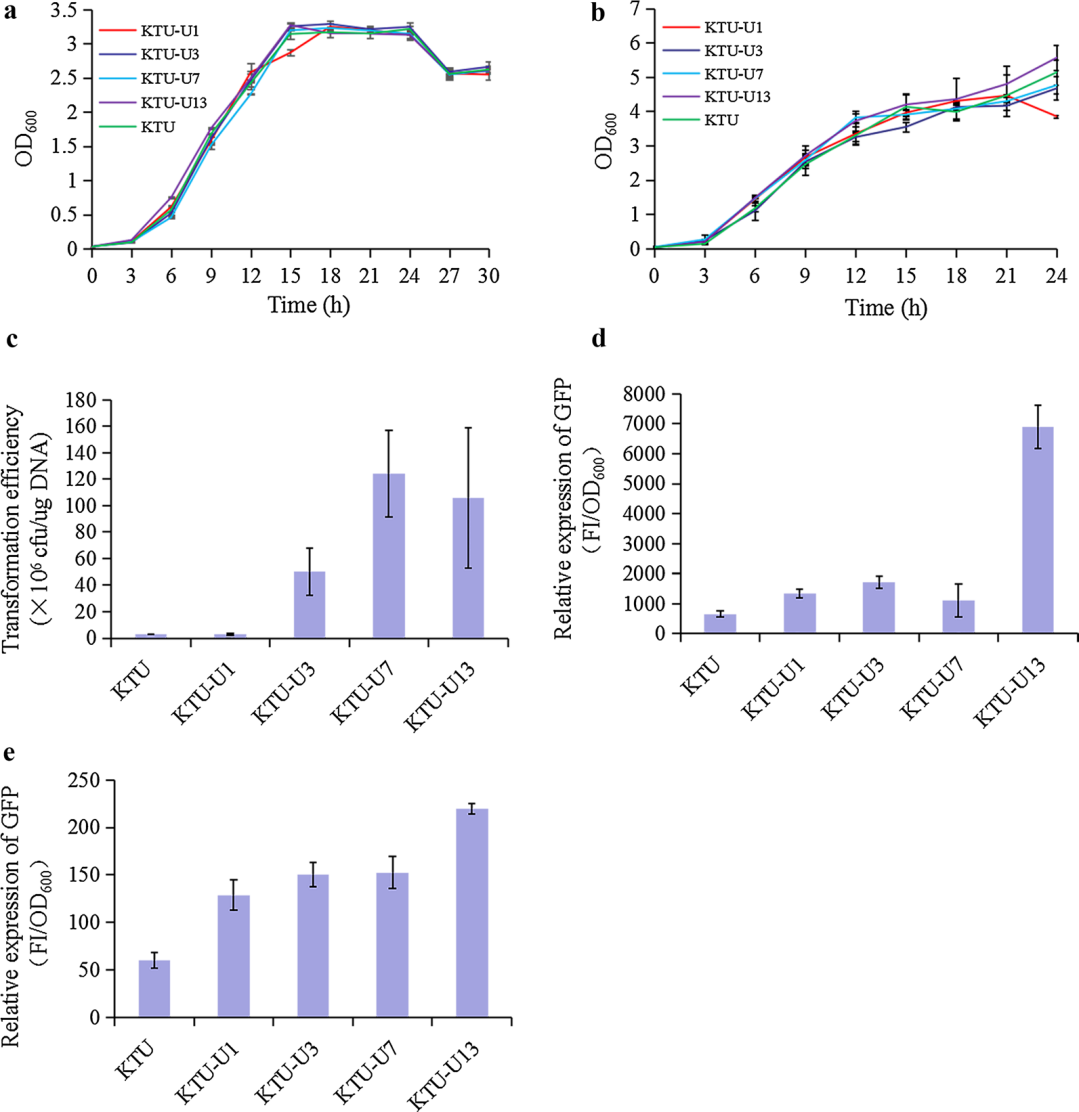
Physiological characteristics assessment of the GIs-deleted mutants of *P. putida* KT2440. **a** Growth curves of the *P. putida* mutants in M9 minimal medium. **b** Growth curves of the *P. putida* mutants in LB medium. **c** The efficiency for transformation of the plasmid pBBR1MCS-2 into the *P. putida* mutants. **d** The relative fluorescence intensities of the *P. putida* mutants grown in M9 minimal medium. **e** The relative fluorescence intensities of the *P. putida* mutants grown in LB medium. *P. putida* KTU was used as a control in all measurements. The data are mean values ± standard deviations from three replicates

Overall, the growth rates of the GIs-deleted mutants showed no significant improvement either in M9G or in LB media compared with the original strain KTU. Moreover, the growth trends of the KT2440 mutants were also very similar to that of the original strain KTU (Fig. 2a, b). These results suggest that the deletion of GIs in the genome may have no significant influence on the growth properties of *P. putida* KT2440.

### Assessment of transformation efficiency and heterologous protein expression capacity

As shown in Fig. 2c, all the KT2440 mutants had different degrees of improvement in the transformation efficiency of plasmid DNA. Especially, the strain KTU-U7 and KTU-U13 had a transformation efficiency of 124.2 × 10^6^ and 105.7 × 10^6^ transformants per µg of plasmid DNA, respectively, which had a 45- and 38-fold improvement in the transformation efficiency compared with the original strain KTU (2.7 × 10^6^ transformants per µg of plasmid DNA). These results suggest that the deletion of GIs may improve the efficiency for transformation of plasmid DNA into *P. putida* KT2440.

GFP was selected as a model protein to study the expression level of heterologous proteins in the KT2440 mutants. As shown in Fig. 2d, e, the relative fluorescence intensities of the GIs-deleted mutants were higher than that of the original strain KTU either in M9G or in LB media. Compared with the original strain KTU, the mutant KTU-U13 showed a 9.4- and 2.7-fold improvement in the relative fluorescence intensity in M9G and LB media, respectively. These results suggest that the deletion of GIs may improve the expression level of heterologous proteins in *P. putida* KT2440.

### Assessment of metabolic activity of the *P. putida* KT2440 mutants

The metabolic phenotypes of the *P. putida* KT2440 mutants were assessed by a Biolog test. There are 71 carbon sources in a Biolog GEN Ш MicroPlate, 22 of which could be utilized by the original strain KTU, 25 by the mutants KTU-U1 and KTU-U3, 49 by the mutant KTU-U7, and 53 by the mutant KTU-U13 (Table 1). The types of monosaccharides utilized by the mutants KTU-U7 and KTU-U13 had increased considerably. Moreover, the capabilities of the KT2440 mutants to utilize certain carbon sources were increasing gradually. The deletion of GIs in the KT2440 genome not only extended the types of substrates utilized by the KT2440 mutants but also improved the capabilities of the KT2440 mutants to utilize the specific substrates.

**Table 1:** Metabolic phenotype analysis of the *P. putida mutants*

Substrate	KTU	KTU-U1	KTU-U3	KTU-U7	KTU-U13
Dextrin	–	–	–	441	625
d-Maltose	–	–	–	433	598
d-Trehalose	–	–	–	438	591
Gentiobiose	–	–	–	295	324
Sucrose	–	–	–	412	600
β-Methyl-d-glucoside	–	–	–	332	576
d-Salicin	–	–	–	288	295
N-Acetyl-d-glucosamine	–	–	–	501	618
N-Acetyl-β-d-mannosamine	–	–	–	–	359
Α-d-Glucose	204	336	345	517	689
d-Mannose	179	200	192	477	701
d-Fructose	–	–	–	544	687
d-Galactose	–	–	–	–	251
d-Fucose	–	–	–	307	433
l-Fucose	–	–	–	–	276
Inosine	–	–	–	482	623
Glycerol	173	374	326	574	723
d-Glucose-6-PO4	–	–	–	463	643
d-Fructose-6-PO	–	–	–	411	637
d-Serine	–	–	–	384	582
Gelatin	–	–	–	378	634
Glycyl-l-prolin	–	–	–	322	247
l-Alanine	241	484	542	652	658
l-Arginine	279	440	440	622	801
l-Aspartic acid	238	617	601	670	770
l-Glutamic	231	672	592	681	824
l-Histidine	381	551	565	662	857
l-Pyroglutamic acid	252	633	585	671	798
l-Serine	–	–	–	537	686
Pectin	–	–	–	365	528
d-Galacturonic acid	213	387	393	510	631
l-Galactonic acid lactone	201	268	323	443	598
d-Gluconic acid	139	451	414	513	761
d-Glucuronic acid	315	471	459	601	665
Glucuronamide	136	192	177	261	376
Mucin acid	169	504	502	614	732
Quinic acid	326	676	677	615	748
d-Saccharic acid	154	493	505	624	702
Methyl pyruvate	–	–	–	427	475
l-Lactic acid	255	423	403	652	836
Citric acid	167	500	571	609	815
α-Keto-glutaric acid	–	146	156	483	578
d-Malic acid	–	–	–	–	346
l-Malic acid	215	437	506	687	836
Bromo-succinic acid	–	–	–	356	228
Tween 40	–	–	–	329	434
γ-Amino-butryric acid	158	433	458	571	730
α-Hydroxy-butyric acid	–	–	–	349	396
β-Hydroxy-d, l butyric acid	166	221	227	538	559
Acetoacetic acid	–	–	–	290	313
Propionic acid	–	342	350	600	664
Acetic acid	–	241	279	562	655
Formic acid	–	–	–	413	620

### Improved PHA production by the GIs-deleted mutant KTU-U13

**Fig. 3:**
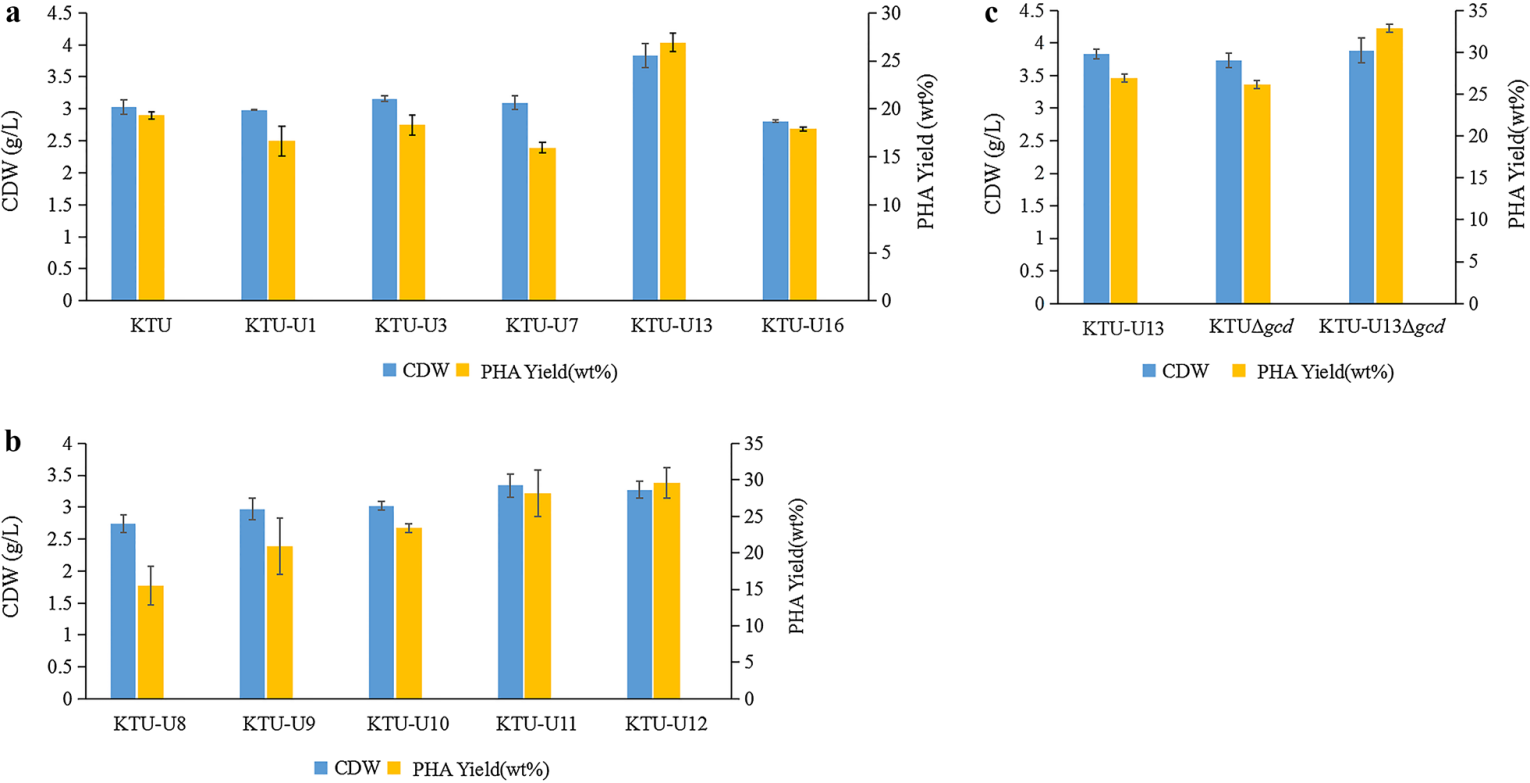
PHA production by the *P. putida* mutants and cell growth of the mutants. **a** Effects of deletion of the GIs on the PHA yield and CDW of the mutants. **b** Determination of the key genomic fragments leading to the improvement in the PHA yield and CDW by stepwise deletion of the five genomic fragments between the mutant KTU-U7 and KTU-U13. **c** Effects of deletion of the glucose dehydrogenase gene *gcd* on the PHA yield and CDW of the mutant KTU-U13. To accumulate PHA, the mutants were incubated in M9 minimal medium supplemented with 20 g/l glucose for 60 h at 30 °C and 180 rpm on a shaker. The data are mean values ± standard deviations from three replicates

The capabilities of the KT2440 mutants to produce PHA were assessed by culturing the mutants in the PHA production medium. As shown in Fig. 3a, the PHA yield and CDW of the final mutant KTU-U13 were improved to 26.93 wt% and 3.83 g/l, which were increased by 39.32% and 26.4%, respectively, compared with the original strain KTU. In order to determine the key factors contributing to the improvement in the PHA yield and CDW of the mutant KTU-U13, the PHA production tests were conducted by using the five mutants between the mutant KTU-U7 and KTU-U13. As shown in Fig. 3b, the PHA yield and CDW of the five mutants were gradually improved with the increase of deleted genomic fragments. The PHA yield of the mutant KTU-U9 was increased by 35.21% compared with the mutant KTU-U8. The PHA yield of the mutant KTU-U10 was increased by 11.75% compared with the mutant KTU-U9. The PHA yield of the mutant KTU-U11 was increased by 20.39% compared with the mutant KTU-U10. Furthermore, the three genomic fragments 9, 10 and 11 were deleted stepwise in the original strain KTU to create the mutant KTU-U16. Unfortunately, the PHA yield and CDW of the mutant KTU-U16 had a significant decrease compared with the mutant KTU-U13 (Fig. 3a). The results from transcriptome analysis showed that the transcriptional levels of both the PHA biosynthesis related genes and central metabolic pathways in the mutant KTU-U13 had no obvious changes compared with the original strain KTU (Additional file 1: Table S3). Finally, we speculate that the improvement in the PHA production capacity of the mutant KTU-U13 may be attributed to the combined effect of the total GIs deleted. The results from scanning electron microscope showed that the deletion of GIs had little effect on cell morphology (Additional file 1: Fig. S2 and Table S4).

Deletion of the glucose dehydrogenase gene *gcd* in *P. putida* KT2440 prevented the formation of gluconate, and most important, increased the PHA yield on glucose by 100% in comparison with its parental strain KT2440 in batch fermentation [22, 23]. In this study, we deleted the *gcd* gene in the mutant KTU-U13. The PHA yield of the mutant KTU-U13Δ*gcd* had a 22.17% increase compared with the mutant KTU-U13 (Fig. 3c), which suggests that the GIs-deleted mutant KTU-U13 may be used as an optimal chassis for enhanced production of PHA by metabolic pathway engineering.

### Deletion of GIs improves the efficiency for the Integration of heterologous pathways into the chromosome of *P. putida*

In this study, the γ-hexachlorocyclohexane (γ-HCH, lindane) and 1,2,3-trichloropropane (TCP) biodegradation pathways were selected for investigating the influence of GIs deletion on the chromosomal integration efficiency of heterologous pathways in *P. putida* KT2440. When using the mutants KTU, KTU-U3 and KTU-U7 as the recipient cells, the desired mutants occurring with the correct pathway integration could almost not be obtained from the selected clones. In contrast, the chromosomal integration efficiencies for the γ-HCH and TCP biodegradation pathways were improved to 50% and 33%, respectively, when using the mutant KTU-U13 as the recipient cell (Table 2).

**Table 2:** Chromosomal integration efficiency of the γ-HCH and TCP biodegradation pathways in the *P. putida* mutants

	KTU	KTU-U3	KTU-U7	KTU-U13	KTUΔ*hsdS*
γ-HCH pathway integration efficiency	1/300^a^	0/48	0/48	48/96	24/48
TCP pathway integration efficiency	1/300	0/48	0/48	16/48	20/48

^a^The ratio of the positive recombinants to the total clones selected

In order to determine the crucial factors for the improvement of the chromosomal integration efficiency of heterologous pathways, we searched for the gene lists of deleted DNA regions between the mutant KTU-U7 and KTU-U13. The *hsdS* gene encoding type I restriction modification system specificity protein was assumed to be a potential candidate contributing to the improvement of the pathway integration efficiency in the mutant KTU-U13. To confirm our hypothesis, we deleted the *hsdS* gene in the control strain KTU, resulting in the mutant KTUΔ*hsdS*. As expected, the chromosomal integration efficiencies for the γ-HCH and TCP biodegradation pathways were 50% and 41%, respectively, when using the mutant KTUΔ*hsdS* as the recipient cell (Table 2).

The biodegradation rates for γ-HCH, TCP and their degradation intermediates were also compared by using the mutants KTU-U13-Lin-TCP and KTU-Lin-TCP. The substrates including γ-HCH, 2,5-dichlorohydroquinone (2,5-DCHQ), TCP and epichlorohydrin (ECH) could be degraded by the two mutants, as shown by GC analysis (Additional file 1: Fig. S3). Interestingly, the mutant KTU-U13-Lin-TCP degraded the substrates faster than did the mutant KTU-Lin-TCP (Additional file 1: Table S5), which suggests that the GIs-deleted mutant KTU-U13 may be an ideal host for enhanced expression of biodegradation pathways.

### The genetic stability of the *P. putida* mutants

**Fig. 4:**
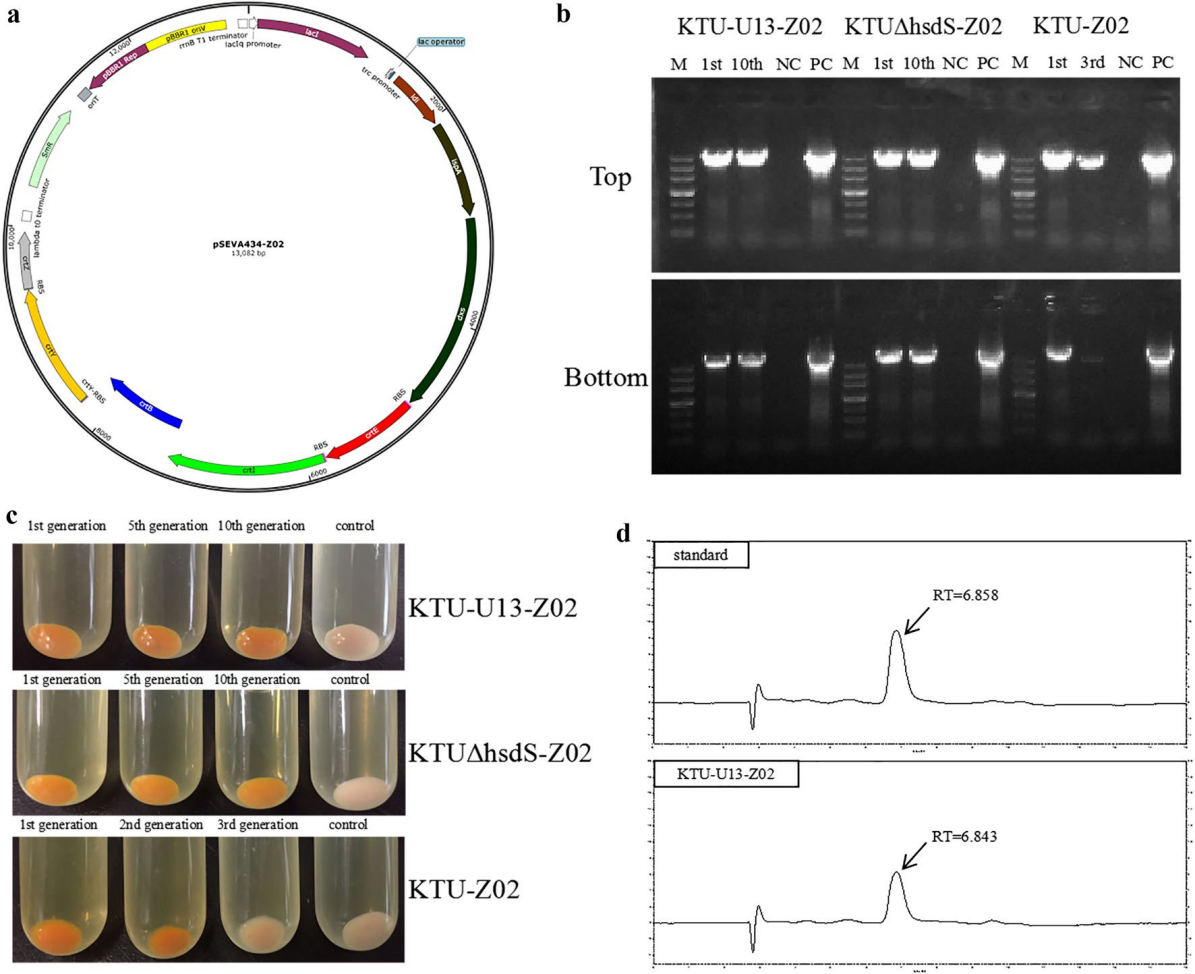
The genetic stability of the *P. putida* mutants transformed with the recombinant plasmid pSEVA434-Z02 containing the intact zeaxanthin biosynthetic pathway. **a** The physical map of the recombinant plasmid pSEVA434-Z02. **b** Detection of the zeaxanthin biosynthetic genes by PCR in the continuous passage cultures of the *P. putida* mutants. Top: detection of the fragment containing the *idi*-*ispA*-*dxs* gene. Bottom: detection of the fragment containing the *crtE*-*crtI*-*crtB*-*crtY*-*crtZ* gene. Symbols: M, DNA marker; 1st, the first generation subculture; 3rd, the third generation subculture; 10th, the tenth generation subculture; NC, the use of ddH_2_O as the template; PC, the use of pSEVA434-Z02 as the template. **c** Test for the capacity of the continuous passage cultures of the *P. putida* mutants to synthesize the yellow product zeaxanthin. The *P. putida* mutants transformed with the empty vector pSEVA434 were used as the negative controls. **d** HPLC analysis for zeaxanthin production by the mutant KTU-U13-Z02

The description of biosynthetic pathway for zeaxanthin is presented in Additional file 1: Fig. S4 [24]. The genetic stability of the mutants KTU, KTU-U13 and KTUΔ*hsdS* was compared by transformation with the plasmid pSEVA434-Z02 carrying zeaxanthin biosynthetic genes (Fig. 4a). The continuous passage cultures of the mutants were detected for the zeaxanthin biosynthetic genes by PCR. As a result, the zeaxanthin biosynthetic genes *idi*, *ispA*, *dxs*, *crtE*, *crtI*, *crtB*, *crtY* and *ctrZ* were still present in the tenth generation subculture of the mutants KTU-U13-Z02 and KTUΔ*hsdS*-Z02, but these genes had been lost in the third generation subculture of the mutant KTU-Z02 (Fig. 4b). During the successive passage cultures of the mutants KTU-U13-Z02 and KTUΔ*hsdS*-Z02, the mutants still retained the capacity to synthesize the yellow product zeaxanthin (Fig. 4c). Zeaxanthin production by the mutant KTU-U13-Z02 was further confirmed by HPLC. A chromatogram peak with a retention time of 6.84 min was detected in HPLC analysis of the cell extract, which was identified as zeaxanthin by comparing with the zeaxanthin standard (Fig. 4d).

## Discussion

### Optimum chassis for constructing the efficient microbial cell factories

A biosafety strain *P. putida* KT2440 is increasingly used as an optimal chassis for heterologous metabolic pathways expression for various biotechnological purposes. The broad metabolic potential of *P. putida* KT2440 was successfully exploited for the functional assembly of novel biosynthetic pathways to synthesize high value-added products as well as for the construction of novel biodegradation pathways to remove environmental pollutants [17, 18, 25, 26]. GIs, which are relatively independent DNA fragments on prokaryotic chromosomes, are usually transferred among different bacterial species through the horizontal gene transfer. According to the function of the genes carried by GIs, GIs can be divided into antibiotic resistance islands, pathogenicity islands, and metabolic islands. Widespread transmission of virulence factors and drug resistance genes may pose a serious threat to human health. The expression of functional genes in metabolic islands may consume unnecessary energy and compete with the endogenous and heterologous metabolic pathways [11, 12]. In this study, 13 GIs, accounting for ~ 4.12% of the total genome size, were identified by the analysis of the complete genome sequence of *P. putida* KT2440 and the putative GIs were successfully knocked out using a scarless large genomic DNA fragment deletion method. By comparing several physiological characteristics such as transformation efficiency, heterologous protein expression, metabolic activity, PHA production capacity, chromosomal integration efficiency and the genetic stability, the GIs-deleted mutant KTU-U13 was demonstrated for its potential to be used as the optimum chassis for constructing the efficient microbial cell factories.

Other researchers previously constructed two genome-reduced strains EM329 and EM383 [7, 8]. Although in this study the GIs were used as deleted targets to generate the mutant KTU-U13, some of the deleted genomic regions are overlap between EM383 and KTU-U13 (Additional file 1: Table S6).

The physiological characteristics of the genome-reduced strains EM329, EM383 and KTU-U13 are summarized in Additional file 1: Table S7. In particular, several important physiological traits had been evaluated for the mutant KTU-U13 but not in previous studies using the mutants EM329 and EM383. Firstly, the mutant KTU-U13 could utilize a wide range of carbon sources, possibly resulting in a higher metabolic diversity and a stronger environmental adaptability. Therefore, the strain KTU-U13 is not only suitable for the heterologous production of target products using a wide variety of substrates, but it also is a promising candidate for bioremediation of contaminated environment. Secondly, the mutant KTU-U13 showed an improvement in the PHA-producing ability, suggesting that the strain may be further engineered for the construction of the efficient PHA producers. Thirdly, the mutant KTU-U13 not noly showed a high efficiency for chromosomal integration of heterologous pathways, but it also was able to stably express plasmid-borne heterologous biosynthetic pathways, which facilitate the implantation of heterologous pathways into the host strain and lay a solid foundation for the development of microbial cell factories. More importantly, the present study reports for the first time the systematic deletion of GIs in a microbial strain to create a superior chassis for synthetic biology applications.

### The improvement of transformation efficiency and heterologous protein expression capacity

Transformation efficiency, which is influenced by cell status, growth stage, nutritional availability, DNA uptake mechanism, and donor DNA concentration and configuration, is considered as one of the critical determinants for genetic manipulation, genome editing and metabolic engineering. In this study, the transformation efficiencies of the GIs-deleted mutants KTU-U7 and KTU-U13 were significantly higher than that of the original strain KTU. We speculate that the knockout of GIs makes the competent cells in the optimal DNA uptake state during electroporation. In this study, by comparing the relative fluorescence intensity of GFP in the original strain KTU and GIs-deleted mutants, it was tentatively proposed that the production capability of foreign proteins had been improved in the GIs-deleted KT2440 mutants.

### The GIs-deleted mutant KTU-U13 may possess a much broader metabolic potential and stronger environmental adaptability

Environmental adaptability is a key factor for a microorganism to grow normally in the environment or develop into dominant bacteria, which largely depends on the utilization capability of this microorganism for various carbon sources. The Biolog phenotype microarray system is a high-throughput technique for measuring microbial metabolic activity [27]. In this study, compared with the original strain KTU, the types of carbon sources utilized by the GIs-deleted mutant KTU-U13 were significantly increased and the metabolic capabilities for many carbon sources were greatly improved, as shown by the Biolog results, which suggest that the GIs-deleted mutant KTU-U13 may possess a much broader metabolic potential and stronger environmental adaptability than the original strain KTU. Therefore, the GIs-deleted mutant KTU-U13 has enormous potential for in situ bioremediation of contaminated environments.

### The improvement in the PHA production capacity of the mutant KTU-U13

Compared with the original strain KTU, the PHA yield and cell dry weight of the GIs-deleted mutant KTU-U13 were increased by 39.32 and 26.4%, respectively. We did not find a clear reason for the improvement of PHA production, either from the changes in the transcriptional level of PHA biosynthesis related genes and central carbon metabolism, or from the consequence of deletion of a specific GI. This improvement in the PHA production capacity may be attributed to a synergic effect of the total GIs deleted, not just an independent effect of deletion of a specific GI. Some GIs may contain certain non-essential metabolic pathways that consume energy. For example, AGI-3 islands found in *E. coli* BEN2908 encode proteins involved in sugar transport and metabolism [28], and RAP29 islands found in *P. aeruginosa* PA2192 encode abietane diterpenoid metabolic pathways [29]. The metabolic islands may consume energy and compete with basal metabolism and the production process of secondary metabolites. In addition, metabolic regulation related genes have been found in certain GIs from bacteria [30]. The GIs deleted in this study may also contain non-essential and high energy-consuming metabolic pathways and certain negative regulatory factors related to PHA biosynthesis. Interestingly, deletion the *gcd* gene had significant effects for improving PHA production in the original strain KTU, but the effects were less pronounced in the GIs-deleted mutant KTU-U13. The differences between KTU and KTU-U13 may be attributed to the efficiencies of gluconate secretion. By search for the gene lists of deleted genomic regions (Additional file 1: Table S2), we found that many membrane proteins and transporter family proteins had been deleted. We assume that the deleted proteins may be related to gluconate secretion. Our assumption had been supported by the fact that the concentrations of D-gluconate in the fermentation media of the GIs-deleted mutants gradually decreased with the reduction of the *P. putida* KT2440 genome (Additional file 1: Fig. S5).

### The restriction-modification system related gene *hsdS* may be crucial for improving the chromosomal integration efficiency of heterologous pathways and genetic stability

Currently, chromosomal integration of heterologous biosynthetic pathways is a common strategy for constructing various industrially producing strains as microbial cell factories. Unfortunately, in most cases, the integration of heterologous genes into the chromosome of host strain is a very inefficient process due to the low transformation efficiency, the presence of unknown restriction-modification systems, and the lack of efficient genetic recombination systems. In this study, compared with the original strain KTU, the integration efficiencies for γ-HCH and TCP biodegradation pathways had about 150- and 100-fold improvement, respectively, in the GIs-deleted mutant KTU-U13. A *hsdRMS* operon coding for a type I DNA restriction-modification system was previously found in *P. putida* KT2440, which typically protect this bacterium against exogenous DNA [7]. In this study, compared with the final mutant KTU-U13, the *hsdS*-deleted mutant KTU showed the same high chromosomal integration efficiency for γ-HCH and TCP biodegradation pathways, suggesting that the deletion of the restriction-modification system related gene *hsdS* may be crucial for significantly improving the chromosomal integration efficiency of heterologous pathways in *P. putida* KT2440.

The GIs-deleted mutant KTU-U13 showed the excellent genetic stability when transformed with the recombinant plasmid pSEVA434-Z02 containing the heterologous zeaxanthin biosynthetic pathway, which was verified by continuous passage culture of the mutant. The tenth generation subculture of the mutant still retained the intact zeaxanthin biosynthetic pathway and it was able to produce zeaxanthin. In contrast, the original strain KTU was unable to maintain the intact zeaxanthin biosynthetic pathway when transformed with the recombinant plasmid pSEVA434-Z02. The successive passage cultures of the *hsdS*-deleted mutant KTU containing the recombinant plasmid pSEVA434-Z02 still maintained an ability to produce zeaxanthin, suggesting that the inactivation of the restriction-modification system related gene *hsdS* may play crucial roles in maintaining the genetic stability of the heterologous biosynthetic pathway in *P. putida* KT2440. The results also suggest that the mutants KTU-U13 and KTUΔ*hsdS* with the superior genetic stability may be ideal hosts for the expression of various heterologous biosynthetic pathways.

## Conclusions

In summary, compared with the original strain KTU, the GIs-deleted mutant KTU-U13 not only shows improvements in many physiological characteristics such as transformation efficiency, heterologous protein expression, carbon source utilization, and chromosomal integration efficiency, but it also has obvious advantages in the production of endogenous products as well as the expression of heterologous metabolic pathways. These desirable traits make the genome-streamlined strain an optimum chassis for synthetic biology applications. This study suggests that the systematic deletion of GIs in various model microorganisms may be a feasible strategy for constructing superior chassis for the development of microbial cell factories.

## Methods

### Chemicals, reagents, bacterial strains, and culture conditions

γ-HCH, 2,5-DCHQ, TCP, and ECH, (99% pure analytical grade) were purchased from Bailingwei Technology Co. Ltd., Beijing, China. The zeaxanthin standard was purchased from Yuanye Bio-Technology Co. Ltd., Shanghai, China. All the other chemicals were purchased from Dingguo Biotechnology Co. Ltd., Tianjin, China. RNA purification kit was purchased from CWBIO Co. Ltd., Beijing, China. All the other molecular biology kits were purchased from Vazyme Biotech Co. Ltd., Nanjing, China.

*Escherichia coli* DH5α was grown at 37 °C in LB medium [31] and used as a host for plasmid construction. *P. putida* KT2440 was used as a starting strain for genome reduction. *P. putida* strains were grown at 30 °C in M9 minimal medium supplemented with 4 g/l glucose (M9G) [19] or LB medium. If necessary, media were supplemented with 50 μg/ml kanamycin (Kan), 50 μg/ml streptomycin (Sm), or 20 μg/ml 5-fluorouracil (5-FU).

### Identification and deletion of GIs in the *P. putida* KT2440 genome

Firstly, 43 GIs were predicted by using a GC-Profile from TUBIC, and the GIs with a 5% difference from the average GC content of the KT2440 genome were selected as the deletion targets. All the strains and plasmids used in this study are listed in Additional file 1: Table S8. All the primers used in this study are listed in Additional file 1: Table S9. The gene knockout vectors for GIs deletions were constructed from the suicide plasmid pK18mobsacB as described previously [32]. Plasmid was transformed into *P. putida* by electroporation using the previously established procedures [33]. The *P. putida* mutants with GIs deletions were constructed based on a scarless genome editing strategy using the suicide plasmid in combination with *upp* as a counter-selectable marker. The detailed procedures for the screening of the single-crossover or double-crossover mutant are described in Gong et al. [32]. All the constructed mutant strains were validated by PCR detection and DNA sequencing.

### Physiological characteristics assessment

The growth characteristics of the *P. putida* KT2440 mutants were measured in LB or M9 minimal medium. Each strain was inoculated at an initial cell density of OD_600_ = 0.02 in 500 ml of shake flasks containing 100 ml of LB or M9 medium supplemented with 0.4% (w/v) glucose and then incubated for 60 h at 30 °C and 180 rpm. The cell growth was estimated by measuring the OD_600_ of the cultures periodically using a UV-1800 spectrophotometer (Shimadzu, Kyoto, Japan).

The efficiency for transformation of plasmid into the *P. putida* KT2440 mutants was determined by electroporation using an *E. coli*-*Pseudomonas* shuttle vector pBBR1MCS-2 [34]. Firstly, the competent cells used for electroporation were prepared according to a standard procedure. Then, 378.4 ng of plasmid DNA was introduced into the competent cells by electroporation. Finally, the cell suspensions were serially diluted and spread on LB agar plates with 50 μg/ml kanamycin. The transformation efficiency was evaluated by calculating the number of colonies grown on the plates.

The production capabilities of heterologous proteins in the *P. putida* KT2440 mutants were evaluated by introducing the plasmid pBBR-*gfp* containing the green fluorescent protein (GFP) gene into the *P. putida* mutants. Firstly, 2 ml of the *P. putida* cultures were withdrawn and centrifuged, and then the cell pellets were washed twice with PBS buffer (pH 7.4) and resuspended in PBS buffer to a final concentration of OD_600_ = 0.5. The fluorescence intensity was measured by using an EnSpire Multimode Reader System (PerkinElmer, Waltham, USA) at an excitation wavelength of 395 nm and an emission wavelength of 509 nm. The relative fluorescence intensity was calculated by normalization against per OD_600_ of whole cells. The fluorescence signal of *P. putida* KT2440 harboring pBBR1MCS-2 was set as background and was subtracted from the overall fluorescence.

The metabolic phenotypes of the *P. putida* KT2440 mutants were determined with a Biolog GEN Ш MicroPlate™ using a phenotype microarray system (Biolog Inc., California, USA). All the tested *P. putida* strains were cultivated in a special medium recommended by Biolog manufacturer. Samples were prepared and estimated according to the manufacturer’s instructions. The bacterial cells on the solid medium surface were collected by cotton swab, dissolved into the inoculating fluid IF-A (Catalog no. 72401), and then the cell density was adjusted to a range of 90–98% Turbidity. Subsequently, 100 μl of samples were inoculated into the Biolog GEN Ш plates with different carbon sources. After the plates were incubated at 30 °C for 24 h, the absorbance at 590 nm for the samples in the 96-well plate was measured with the Biolog reader and the test data were analyzed by the Biolog system.

### Transcriptome analysis

The *P. putida* KTU and KTU-U13 were cultivated in M9G, and then the bacterial cultures were sampled after incubation for 12 h. Subsequently, the total RNA was extracted from the samples using an RNApure bacteria kit (Cwbio, Beijing, China). Qualified samples with RIN larger than 8 were submitted to Majorbio (Shanghai, China) for RNA-seq. Transcriptome analysis was carried out by using a standard pipeline. The detailed analysis process referred to a previous literature [35].

### Scanning electron microscope

The samples used for scanning electron microscope were prepared as described previously [36]. Finally, the prepared samples were coated with gold and observed using a Quanta 200 scanning electron microscope (FEI, Hillsboro, USA) for imaging.

### PHA production by *P. putida*

*P. putida* strains were pre-cultured in 500 ml shake flasks containing 100 ml of LB medium for 12 h at 30 °C and 180 rpm on a shaker. Then, cells were harvested by centrifugation, washed twice with M9 minimal medium, and resuspended with 10 ml of M9 minimal medium. To intracellularly accumulate PHA granules, the cell suspensions were further inoculated into 500-ml shake flasks containing 100 ml of M9 minimal medium supplemented with 20 g/l glucose, and then the samples were incubated for 60 h at 30 °C and 180 rpm on a shaker. After the fermentation was completed, the bacterial cells were collected by centrifugation at 13,000*g* and 4 °C for 20 min, lyophilized and weighed. PHA extraction and purification were performed according to the previous methods [35].

### Chromosomal integration of the γ-HCH and TCP biodegradation pathways

Construction of the γ-HCH and TCP biodegradation pathways was carried out by using the previously developed DNA assembler method, which relies on the efficient homologous recombination mechanism of *Saccharomyces cerevisiae* [37]. Since an overlap region between two adjacent pathway fragments was generated by PCR primer design, cotransformation of the pathway fragments and a linearized helper plasmid pRS416 will allow them to be assembled into a single circular DNA molecule in *S. cerevisiae* through homologous recombination. The isolated plasmid was subsequently introduced by transformation into *E. coli* BW25141 for plasmid enrichment and verification. The assembled pathway was subcloned into the suicide plasmid pK18mobsacB [38] to generate the targeting vector. For chromosomal integration of the γ-HCH and TCP biodegradation pathways, the targeting vector was introduced by electroporation into the *P. putida* strains. Subsequently, the screening of the correct mutants was performed by using a kanamycin resistant marker followed by use of *upp* as a counter-selectable marker. The γ-HCH and TCP biodegradation experiments and gas chromatography (GC) analysis were performed as described previously [32, 39].

### The genetic stability study of the *P. putida* mutants

The recombinant plasmid pSEVA434-Z02 containing the intact zeaxanthin biosynthetic pathway was constructed by our lab and introduced into the mutants KTU, KTUΔ*hsdS* and KTU-U13 by electroporation. The yellow colonies appeared on LB agar plates with streptomycin were inoculated into 5 ml LB medium with streptomycin and the samples were then cultivated at 30 °C for 12 h. The continuous passage cultures were repeated 10 times in 5 ml LB medium with streptomycin. To evaluate the genetic stability of the passage cultures, the zeaxanthin biosynthetic genes and zeaxanthin production were detected by PCR and HPLC, respectively. Zeaxanthin production by *P. putida*, zeaxanthin extraction and HPLC analysis were performed according to the previous reports [24, 40].

## Supplementary information

**Additional file 1.** Additional figures and tables.

## Data Availability

All data generated or analyzed during this study are included in this published article and its additional file.
