# Supplementary material for: Deletion of genomic islands in the Pseudomonas putida KT2440 genome can create an optimal chassis for synthetic biology applications
Source: Microb Cell Fact. 2020 Mar 18;19:70. doi: 10.1186/s12934-020-01329-w (PMC7081699; doi:10.1186/s12934-020-01329-w)
Supplement: Supplementary file 1 — Additional file 1. Additional figures and tables. [file 12934_2020_1329_MOESM1_ESM.doc]

**Additional material**

**Deletion of genomic islands in the *Pseudomonas putida* KT2440 genome can create an optimal chassis for synthetic biology applications**

Peixin Liang,^1^ Yiting Zhang,^1^ Bo Xu,^1^ Yuxin Zhao,^1^ Xiangsheng Liu,^1^ Weixia Gao,^1^ Ting Ma,^1^ Chao Yang,^1^* Shufang Wang,^2^* Ruihua Liu ^2^*

^1^Key Laboratory of Molecular Microbiology and Technology for Ministry of Education, Nankai University, Tianjin 300071, China

^2^State Key Laboratory of Medicinal Chemical Biology, Nankai University, Tianjin 300071, China

*Correspondence to:

Chao Yang (Tel./Fax: +86 22 23503866, E-mail: yangc20119@nankai.edu.cn)

Shufang Wang (Tel./Fax: +86 22 23503753; E-mail: wangshufang@nankai.edu.cn)

Ruihua Liu (Tel./Fax: +86 22 23502351, E-mail: yangyangliu@nankai.edu.cn)

**Table S1** Deleted genomic regions in the construction processes of the GIs-deleted *P*. *putida* mutants

| Deleted region | Position (start-end) | Size of deleted DNA fragment (bp) |
| --- | --- | --- |
| region 1 | 4173205-4235064 | 61859 |
| region 2 | 6123617-6170294 | 46677 |
| region 3 | 4483000-4501061 | 18061 |
| region 4 | 5002215-5025579 | 23364 |
| region 5 | 5062618-5075236 | 12618 |
| region 6 | 2009003-2021463 | 12460 |
| region 7 | 4388784-4398856 | 10072 |
| region 8 | 4618245-4632395 | 14150 |
| region 9 | 4148822-4157878 | 9056 |
| region 10 | 3496831-3522213 | 25382 |
| region 11 | 2816532-2822315 | 5783 |
| region 12 | 5396988-5403702 | 6714 |
| region 13 | 2163139-2171465 | 8326 |

**Table S2** Gene annotation of deleted genomic regions in *P. putida*

| Deletion units | Name of gene | Position (start-end) | Product |
| --- | --- | --- | --- |
| region 1 | unknown  unknown  unknown  unknown  unknown  unknown  unknown  unknown  unknown  unknown  unknown  unknown  unknown  unknown  unknown  unknown  unknown  unknown  unknown  unknown  unknown  unknown  unknown  unknown  unknown  unknown  unknown  unknown  unknown  unknown  unknown  unknown  unknown  unknown  unknown  unknown  unknown  unknown  unknown  unknown  unknown  unknown  unknown  unknown  unknown  unknown  unknown  unknown  unknown  unknown  unknown  unknown | [4173205:4173778](-)  [4175319:4177431](+)  [4177427:4179614](+)  [4179594:4180143](+)  [4180135:4181599](+)  [4181661:4181985](-)  [4182222:4184226](+)  [4184222:4185881](+)  [4185931:4186084](+)  [4186653:4187895](+)  [4188104:4188404](+)  [4188737:4189556](+)  [4189763:4189946](+)  [4190056:4190668](-)  [4191824:4192394](+)  [4193042:4193498](+)  [4193647:4193959](+)  [4194147:4194552](-)  [4194563:4194782](-)  [4194974:4195673](-)  [4195711:4196158](+)  [4196526:4196769](-)  [4197148:4197682](-)  [4198165:4199173](+)  [4199714:4201037](-)  [4201151:4207775](-)  [4208047:4210723](+)  [4210791:4211076](-)  [4211125:4211491](-)  [4211655:4212615](-)  [4212572:4213724](-)  [4213730:4214951](-)  [4214957:4217042](-)  [4217038:4217794](-)  [4217796:4219296](-)  [4219434:4221141](+)  [4221402:4222509](-)  [4222650:4222854](-)  [4222868:4223036](-)  [4223093:4223888](-)  [4224708:4224924](-)  [4224924:4226097](-)  [4226216:4226897](-)  [4226883:4227843](-)  [4227857:4228256](-)  [4228791:4230207](-)  [4230227:4231265](-)  [4231380:4231740](-)  [4231823:4232114](-)  [4232331:4232793](-)  [4232861:4233323](+)  [4233552:4235064](-) | hypothetical protein  hypothetical protein  hypothetical protein  hypothetical protein  hypothetical protein  hypothetical protein  hypothetical protein  putative Helicase  hypothetical protein  hypothetical protein  hypothetical protein  transcriptional regulator  hypothetical protein  hypothetical protein  hypothetical protein  hypothetical protein  hypothetical protein  hypothetical protein  hypothetical protein  hypothetical protein  Cro/CI family transcriptional regulator  hypothetical protein  hypothetical protein  serine/threonine protein phosphatase  hypothetical protein  DNA helicase-related protein  hypothetical protein  hypothetical protein  transcriptional regulator MvaT  hypothetical protein  hypothetical protein  hypothetical protein  hypothetical protein  hypothetical protein  hypothetical protein  hypothetical protein  chromosome partitioning ATPase  hypothetical protein  hypothetical protein  hypothetical protein  hypothetical protein  membrane protein  membrane protein  hypothetical protein  hypothetical protein  hypothetical protein  hypothetical protein  hypothetical protein  hypothetical protein  hypothetical protein  repressor-like DNA-binding domain-containing protein  diguanylate cyclase |
| region 2 | unknown  unknown  unknown  unknown  unknown  unknown  copB-II  unknown  copA-II  unknown  unknown  copR-II  copS  unknown  czcC  cusB  cusA  cusF  unknown  unknown  unknown  unknown  unknown  unknown  unknown  unknown  unknown  unknown  unknown  unknown  unknown  unknown  unknown  unknown  unknown  unknown  unknown  unknown  unknown  unknown  unknown  unknown  unknown  unknown | [6123617:6124808](-)  [6125205:6126909](+)  [6126975:6127884](-)  [6128805:6129138](+)  [6129204:6129660](-)  [6129649:6130432](-)  [6130441:6131521](-)  [6131510:6131828](-)  [6131842:6133852](-)  [6134047:6134302](+)  [6134433:6134793](-)  [6134985:6135663](+)  [6135659:6137069](+)  [6137105:6137456](+)  [6137545:6138799](+)  [6138795:6140268](+)  [6140264:6143423](+)  [6143419:6143761](+)  [6143972:6144464](-)  [6144557:6144755](+)  [6144779:6145439](-)  [6145435:6145711](-)  [6145736:6146318](-)  [6146327:6147350](-)  [6147615:6147816](-)  [6150989:6151835](+)  [6151827:6152403](+)  [6152419:6152908](+)  [6152938:6154474](-)  [6154537:6154873](-)  [6154869:6155190](-)  [6155395:6156166](-)  [6156560:6157628](+)  [6157636:6158278](+)  [6158384:6158663](+)  [6159530:6160319](-)  [6160526:6161183](+)  [6161241:6161538](+)  [6161592:6161904](+)  [6161926:6163273](-)  [6163269:6165282](-)  [6165278:6166241](-)  [6166920:6168519](+)  [6169112:6170294](-) | Rieske 2Fe-2S family protein  choline/carnitine/betaine transporter family protein  LysR family transcriptional regulator  hypothetical protein  hypothetical protein  metal-binding protein  copper resistance protein B  metal-binding protein  copper resistance protein A  hypothetical protein  hypothetical protein  transcriptional regulator  copper sensor protein  metal-binding protein  CzcC family metal RND transporter outer membrane protein  CzcB family copper RND transporter membrane fusion protein  CzcA family RND family copper transporter  copper efflux protein  hypothetical protein  hypothetical protein  hypothetical protein  hypothetical protein  hypothetical protein  WD40/YVTN repeat-containing protein  metal-binding chaperone  hypothetical protein  hypothetical protein  hypothetical protein  transposase  transposase  transposase  hypothetical protein  hypothetical protein  hypothetical protein  hypothetical protein  NAD-dependent acetyl-CoA synthetase deacetylase subunit  hypothetical protein  hypothetical protein  hypothetical protein  TnsC-likehypothetical proteinn  transposase  transposase  TnsD-like transposition protein  hypothetical protein |
| region 3 | unknown  unknown  unknown  unknown  unknown  unknown  unknown  unknown  unknown  unknown  unknown  unknown  unknown  unknown  unknown  unknown | [4483000:4483462](-)  [4483669:4484356](+)  [4484824:4486126](+)  [4486128:4486299](+)  [4486395:4486716](+)  [4486712:4487048](+)  [4487111:4488647](+)  [4489217:4491863](-)  [4492117:4492900](+)  [4493378:4493639](-)  [4493707:4495240](-)  [4495258:4495615](-)  [4495690:4496650](+)  [4496701:4497949](-)  [4497954:4500003](-)  [4500014:4501061](-) | hypothetical protein  isochorismatase family hydrolase  hypothetical protein  hypothetical protein  transposase  transposase  transposase  hypothetical protein  hypothetical protein  transposase  transposase  transposase  site-specific recombinase  hypothetical protein  hypothetical protein  DNA-cytosine methyltransferase |
| region 4 | unknown  unknown  unknown  unknown  unknown  unknown  unknown  unknown  unknown  unknown  unknown  unknown  insN  unknown  unknown  unknown  gabD-II  unknown  unknown  unknown  unknown  unknown  unknown  unknown | [5002215:5003646](+)  [5003648:5006948](+)  [5007018:5007618](+)  [5008516:5009065](+)  [5009061:5009640](+)  [5009811:5010132](+)  [5010490:5011591](+)  [5011692:5011917](+)  [5012006:5012408](-)  [5012419:5012638](-)  [5013179:5013470](+)  [5013564:5013888](+)  [5014189:5014498](+)  [5014542:5015352](+)  [5015524:5015737](-)  [5015970:5017362](-)  [5017383:5018856](-)  [5019115:5020111](-)  [5020313:5020781](+)  [5020831:5021599](-)  [5021697:5022354](-)  [5022353:5023010](-)  [5023080:5023935](-)  [5024178:5025579](+) | site-specific recombinase  hypothetical protein  hypothetical protein  hypothetical protein  hypothetical protein  hypothetical protein  hypothetical protein  hypothetical protein  hypothetical protein  hypothetical protein  hypothetical protein  hypothetical protein  transposase  transposase  hypothetical protein  aminotransferase  succinate-semialdehyde dehydrogenase  hypothetical protein  HTH-type transcriptional regulator  amino acid ABC transporter ATP-binding protein  amino acid ABC transporter permease  amino acid ABC transporter permease  amino acid ABC transporter substrate-binding protein  GntR family transcriptional regulator |
| region 5 | unknown  unknown  unknown  unknown  unknown  unknown  unknown  unknown  unknown  unknown  unknown  unknown  unknown | [5062618:5063545](+)  [5063703:5064996](-)  [5065106:5065772](-)  [5065768:5066590](-)  [5066630:5067536](-)  [5067951:5069304](-)  [5070013:5070886](-)  [5071131:5072034](+)  [5072280:5072850](-)  [5073231:5073615](-)  [5073674:5074121](-)  [5074170:5074563](-)  [5074744:5075236](+) | LysR family transcriptional regulator  MFS transporter  4-hydroxy-4-methyl-2-oxoglutarate aldolase  carbon-nitrogen hydrolase family protein  LysR family transcriptional regulator  Porin  TauD/TfdA family dioxygenase  LysR family transcriptional regulator  hypothetical protein  hypothetical protein  hypothetical protein  hypothetical protein  Cro/CI family transcriptional regulator |
| region 6 | unknown  unknown  unknown  unknown  unknown  unknown | [2009003:2011433](-)  [2011432:2014021](-)  [2014044:2015487](-)  [2015504:2016392](-)  [2018725:2020051](+)  [2020047:2021463](+) | group 2 family glycosyl transferase  group 2 family glycosyl transferase  hypothetical protein  hypothetical protein  HlyD family secretion protein  outer membrane efflux protein |
| region 7 | unknown  unknown  unknown  unknown  unknown  unknown  unknown  unknown  unknown  unknown  unknown  unknown  unknown | [4388784:4390740](-)  [4390732:4390924](-)  [4390869:4391166](-)  [4391162:4391516](-)  [4391592:4393014](-)  [4393483:4394971](-)  [4394970:4395174](-)  [4395179:4395773](-)  [4395802:4396318](-)  [4396310:4396685](-)  [4396687:4397239](-)  [4397241:4397568](-)  [4397542:4398856](-) | tail protein  hypothetical protein  hypothetical protein  tail tube protein  group II intron-encoding maturase  sheath protein  FluMu protein gp38  hypothetical protein  hypothetical protein  hypothetical protein  hypothetical protein  hypothetical protein  hypothetical protein |
| region 8 | unknown  unknown  unknown  unknown  unknown  unknown  unknown | [4618245:4618731](+)  [4620990:4621407](+)  [4625970:4627503](-)  [4627521:4627881](-)  [4629581:4629728](+)  [4629740:4630226](+)  [4631660:4632395](+) | hypothetical protein  Phosphotriesterase  transposase  transposase  hypothetical protein  hypothetical protein  hypothetical protein |
| region 9 | unknown  unknown  unknown  unknown  unknown  unknown  unknown  unknown | [4148822:4149437](-)  [4149548:4150001](+)  [4150397:4151810](+)  [4151960:4153223](-)  [4153383:4154058](-)  [4154140:4155493](-)  [4155764:4156790](-)  [4156990:4157878](-) | LysE family transporter  leucine-responsive regulatory protein  cytosine/purine/uracil/thiamine/allantoin permease family protein  aromatic compound-specific porin  p-nitrobenzoate reductase NfnB  aromatic compound MFS transporter  transcriptional regulator, AraC family  LysR family transcriptional regulator |
| region 10 | unknown  unknown  unknown  unknown  unknown  unknown  unknown  unknown  unknown  unknown  unknown  unknown  unknown  unknown  unknown  unknown  unknown  unknown  unknown  unknown  unknown  unknown  unknown  unknown  unknown  unknown  unknown  unknown | [3496831:3497596](-)  [3498301:3498853](-)  [3500052:3500325](-)  [3500541:3500781](-)  [3501266:3501458](+)  [3501613:3502312](+)  [3502345:3502549](+)  [3502703:3503015](-)  [3503023:3503305](-)  [3504008:3504185](-)  [3504274:3504571](-)  [3504567:3505641](-)  [3506121:3506346](-)  [3506682:3507651](+)  [3507651:3508347](+)  [3508372:3510229](+)  [3510239:3510824](+)  [3510804:3511326](+)  [3511332:3515490](+)  [3515483:3515960](+)  [3517094:3517625](+)  [3517883:3518315](+)  [3518373:3518853](+)  [3519153:3519381](+)  [3520260:3520728](+)  [3521487:3521847](+)  [3521806:3522019](+)  [3522027:3522213](+) | hypothetical protein  membrane protein  hypothetical protein  hypothetical protein  hypothetical protein  membrane protein  hypothetical protein  hypothetical protein  hypothetical protein  hypothetical protein  hypothetical protein  hypothetical protein  hypothetical protein  hypothetical protein  membrane protein  hypothetical protein  hypothetical protein  hypothetical protein  rhs family protein  hypothetical protein  hypothetical protein  hypothetical protein  hypothetical protein  hypothetical protein  hypothetical protein  hypothetical protein  hypothetical protein  hypothetical protein |
| region 11 | unknown  unknown  unknown  unknown  unknown  unknown | [2816532:2816889](+)  [2817202:2818045](-)  [2818649:2818904](+)  [2819230:2820199](+)  [2820710:2821331](-)  [2821721:2822315](+) | MerR family transcriptional regulator  hypothetical protein  transcriptional regulator  hypothetical protein  glutathione S-transferase family protein  TetR family transcriptional regulator |
| region 12 | hsdS  unknown  unknown  unknown  unknown | [5396988:5398719](+)  [5398729:5399158](+)  [5399187:5400624](+)  [5401791:5403324](-)  [5403342:5403702](-) | type I restriction modification system specificity protein  hypothetical protein  hypothetical protein  transposase  transposase |
| region 13 | unknown  unknown  unknown  unknown  unknown  unknown  unknown | [2163139:2164153](+)  [2164316:2165537](-)  [2165604:2166156](-)  [2166936:2168100](+)  [2168787:2169636](-)  [2169739:2170528](-)  [2170658:2171465](+) | hypothetical protein  hypothetical protein  hypothetical protein  hypothetical protein  hypothetical protein  hypothetical protein  hypothetical protein |

**Table S3** Expression profiles of the genes belonging to PHA biosynthesis and central metabolic pathways in *P. putida* strain KTU-U13 and KTU

| Gene name | Locus tag | Description | Log 2 change  (KTU-U13 vs. KTU) |
| --- | --- | --- | --- |
| PHA synthesis |  |  |  |
| *phal* | PP5008 | PHA granule-associate | -0.80 |
| *phaF* | PP5007 | PHA granule-associate | -0.48 |
| *phaC1(phaA)* | PP5003 | PHA polymerase | -0.10 |
| *phaC2* | PP5005 | PHA polymerase | -0.27 |
| *phaZ (phaB)* | PP5004 | PHA depolymerase | -0.34 |
| *phaD* | PP5006 | Transcriptional regulator | 0.05 |
| *phaG* | PP1408 | Acyl-transferase | 0.06 |
| *Transporters* |  |  |  |
| *oprB-1* | PP1019 | Porin | -0.87 |
| *oprB-2* | PP1445 | Porin | -0.38 |
| Glycolysis/gluconeogeneis |  | | |
| *glk* | PP1011 | Glucokinase | -0.06 |
| *pgi* | PP1808 | Glucose-6-phosphate isomerase | 0.24 |
| *fba* | PP4960 | Fructose-1,6-bisphosphate aldolase | -0.24 |
| *tpiA* | PP4715 | Triose phosphate isomerase | -0.40 |
| *gap1(gapA)* | PP1009 | GAP dehydrogenase,typeⅠ | 0.04 |
| *gap2(gapB)* | PP2149 | GAP dehydrogenase,typeⅡ | -0.13 |
| *pgk* | PP4963 | Phosphoglycerate kinase | -0.25 |
| *Pgm* | PP5056 | Phosphoglyceromutase | 0.01 |
| *eno* | PP1612 | Phosphopyruvate hydratase | -0.13 |
| *pyk* | PP1362 | Pyruvate kinase | -0.04 |
| Pentose phosphate pathways |  |  |  |
| *zwf1* | PP1022 | G6P dehydrogenase | -0.45 |
| *zwf2* | PP4042 |  | 0.15 |
| *zwf3* | PP5351 |  | 0.30 |
| *pgl* | PP1023 | 6-phosphogluconate dehydrogenase | -0.38 |
| *Gnd* | PP4043 | 6-phosphogluconate dehydrogenase | 0.36 |
| *gnuK* | PP3416 | Carbohydrate kinase | -0.26 |
| *kguK* | PP3378 | Dehydroglucokinase | -0.08 |
| *kguD* | PP3376 | 2-Ketogluconate 6-phosphate reductase | 0.045 |
| *rpiA* | PP5150 | Ribose-5-phosphate isomerase A | -0.22 |
| *rpe* | PP0415 | Ribulose-phosphate 3-epimerase | -0.16 |
| *tktA* | PP4965 | Transketolase | -0.13 |
| *tal* | PP2168 | Transaldolase B | -0.05 |
| Entner-Doudoroff pathway |  |  |  |
| *edd* | PP1010 | 6-Phosphogluconate dehydratase | -0.09 |
| *eda* | PP1024 | KDPG aldolase | -0.28 |
| Pyruvate metabolism |  |  |  |
| *acoA* | PP0555 | Pyruvate dehydrogenase | -0.08 |
| *acoB* | PP0554 | Pyruvate dehydrogenase | 0.12 |
| *acoC* | PP0553 | Pyruvate dehydrogenase | 0.63 |
| *aldB-I* | PP0545 | Aldehyde dehydrogenase | 0.39 |
| *acsA* | PP4487 | Acetyl-CoA synthetase | -0.20 |
| *accC-2* | PP5347 | Pyruvate carboxylase | -0.39 |
| *ppsA* | PP2082 | Phosphoenolpyruvate synthase | -0.07 |
| *ppc* | PP1505 | Phosphoenolpyruvate carboxylase | 0.28 |
| TCA cycle |  |  |  |
| *gltA* | PP4194 | Citrate synthase | 0.36 |
| *acnA* | PP2112 | Aconitate hydratase | 0.15 |
| *acnB* | PP2339 | Aconitate hydratase | 0.13 |
| *icd* | PP4011 | Isocitrate dehydrogenase | 0.28 |
| *sucA* | PP4189 | 2-Oxoglutarate dehydrogenase | 0.04 |
| *sucD* | PP4185 | Succinyl-CoA synthetase sub alpha | -0.15 |
| *sucC* | PP4186 | Succinyl-CoA synthetase sub beta | -0.15 |
| *sdhA* | PP4191 | Succinate dehydrogenase | -0.07 |
| *fumC* | PP0944 | Fumarate hydratase | 0.09 |
| *mdh* | PP0654 | Malate dehydrogenase | 0.57 |
| Glyoxylate shunt |  |  |  |
| *aceA*  *glcB* | PP4116  PP0356 | Isocitrate lyase  Malate synthase | 1.11  -0.08 |

**Table S4** Cell size of the original strain KTU and the GIs-deleted mutant KTU-U13 in LB medium

| Strains | Average cell length (μm) | Average cell width (μm) |
| --- | --- | --- |
| KTU-U13 | 1.35 ± 0.068 | 0.59 ± 0.001 |
| KTU | 1.22 ± 0.124 | 0.55 ± 0.046 |

**Table S5** Substrate degradation efficiencies of two *P. putida* mutants

| Strain | γ-HCH | 2,5-DCHQ | TCP | ECH |
| --- | --- | --- | --- | --- |
| KTU-Lin-TCP | 23.24%^a^ | 26.40%^b^ | 43.51%^c^ | 14.48%^d^ |
| KTU-U13-Lin-TCP | 43.44% | 32.85% | 58.11% | 31.56% |

a. γ-HCH degradation efficiency within 24 h

b. 2,5-DCHQ degradation efficiency within 1 h

c. TCP degradation efficiency within 24 h

d. ECH degradation efficiency within 1 h

**Table S6** The overlap regions of deleted fragments in genome-reduced strains KTU-U13 and EM383

| Deleted region of KTU-U13 (position: start-end) | Deleted region of EM383  (position: start-end) | The overlap region  (position: start-end) |
| --- | --- | --- |
| region 2 (6123617-6170294) | Tn7 transposase (6161914-6168509) | 6161914-6170294 |
| region 7 (4388784-4398856) | Prophage 1 (4372649-4427414) | 4388784-4398856 |
| region 12 (5396988-5403702) | hsdRMS (5393057-5398717) | 5396988-5398717 |

**Table S7** The physiological characteristics of the genome-reduced strains EM329, EM383 and KTU-U13

| Physiological characteristics | EM329 | EM383 | KTU-U13 |
| --- | --- | --- | --- |
| Growth parameters | The mutant strain had a shortened lag phase being more evident on fructose; the growth rate of the mutant strain was significantly lower in LB. | The mutant strain had a significantly shorter lag phase; the multiple deletions introduced do not significantly affect the growth performance of strain EM383; the OD_600_ values of the mutant strain both in LB medium and in M9 minimal medium amended with fructose were remarkably higher. | The deletion of GIs in the genome had no significant influence on the growth properties. |
| Heterologous gene expression | The mutant strain had a higher yield of recombinant protein (*Y_GFP/X_*). | The mutant strain had a higher yield of recombinant protein (*Y_GFP/X_*). | The deletion of GIs improved the expression level of heterologous proteins. |
| Plasmid stability | The deletion of genes in the genome had no significant influence on the plasmid stability. | The mutant strain reflected a higher stability of extra-chromosomal DNA. | The mutant strain showed the excellent genetic stability when transformed with the recombinant plasmid pSEVA434-Z02 containing the heterologous zeaxanthin biosynthetic pathway. |
| Plasmid transfer efficiency | ND | Electroporation: the mutant strain maintained its transformation capacity within the same order of magnitude known for the wild-type bacterium; mating: the mutant strain had a significantly higher capacity of plasmid acquisition through conjugal delivery. | The mutant strain had a significant improvement in the transformation efficiency. |
| Adenylate energy charge (AEC) | The mutant strain had a significantly higher AEC, and this parameter reflects enhanced anabolic capability. | The mutant strain had a significant increase in the NADPH/NADP^+^ ratio, and such redox charge was manifested mostly for anabolic processes. | ND |
| Endogenous oxidative stress | The mutant strain had a higher reducing power level to rapidly cope with oxidative stressors. | The mutant strain showed a high resistance to the oxidative agent. | ND |
| Cellular viability | Cultures of the mutant strain had fewer dead cells in LB. | Cultures of the mutant strain had fewer dead cells in LB. | ND |
| Biomass coefficients (*Y_X/S_* value) | The mutant strain showed a higher *Y_X/S_* value. | The mutant strain showed a higher *Y_X/S_* value. | ND |
| *m*_s_ values（the maintenance coefficient） | The mutant strain had a lower *m*_s_ value. | The mutant strain had a lower *m*_s_ value. | ND |
| Metabolic activity | ND | ND | The deletion of GIs not only extended the types of substrates utilized but also improved the capabilities to utilize the specific substrates. |
| Capabilities to produce PHA | ND | ND | The PHA yield and CDW of the mutant strain KTU-U13 were improved. |
| Chromosomal integration efficiency | ND | ND | The chromosomal integration efficiencies of the mutant strain KTU-U13 were improved. |

ND, not detected.

**Table S8** Bacterial strains and plasmids used in this study

| Strains or plasmids | Relevant characteristics | Source or reference |
| --- | --- | --- |
| Strains |  |  |
| *E. coli* DH5α | Δ(*lacZYA-argF*)U169,*recA*1,*endA*1, *phoA*, *supE*44, *thi-*1, *relA*1 | Takara |
| *P. putida* KT2440 | wild typ | ATCC 47054 |
| KTU | *upp*-deficient KT2440 | This lab |
| KTU-U1 | KT2440 mutant (Δ*upp*, Δregion 1) | This study |
| KTU-U2 | KT2440 mutant (Δ*upp*, Δregion 1, Δregion2) | This study |
| KTU-U3 | KT2440 mutant (Δ*upp*, Δregion 1, Δregion 2, Δregion3) | This study |
| KTU-U4 | KT2440 mutant (Δ*upp*, Δregion 1, Δregion 2, Δregion 3, Δregion 4) | This study |
| KTU-U5 | KT2440 mutant (Δ*upp*, Δregion 1, Δregion 2, Δregion 3, Δregion 4, Δregion 5) | This study |
| KTU-U6 | KT2440 mutant (Δ*upp*, Δregion 1, Δregion 2, Δregion 3, Δregion 4, Δregion 5, Δregion 6) | This study |
| KTU-U7 | KT2440 mutant (Δ*upp*, Δregion 1, Δregion 2, Δregion 3, Δregion 4, Δregion 5, Δregion 6, Δregion 7) | This study |
| KTU-U8 | KT2440 mutant (Δ*upp*, Δregion 1, Δregion 2, Δregion 3, Δregion 4, Δregion 5, Δregion 6, Δregion 7, Δregion 8) | This study |
| KTU-U9 | KT2440 mutant (Δ*upp*, Δregion 1, Δregion 2, Δregion 3, Δregion 4, Δregion 5, Δregion 6, Δregion 7, Δregion 8, Δregion 9) | This study |
| KTU-U10 | KT2440 mutant (Δ*upp*, Δregion 1, Δregion 2, Δregion 3, Δregion 4, Δregion 5, Δregion 6, Δregion 7, Δregion 8, Δregion 9, Δregion 10) | This study |
| KTU-U11 | KT2440 mutant (Δ*upp*, Δregion 1, Δregion 2, Δregion 3, Δregion 4, Δregion 5, Δregion 6, Δregion 7, Δregion 8, Δregion9, Δregion 10, Δregion 11) | This study |
| KTU-U12 | KT2440 mutant (Δ*upp*, Δregion 1, Δregion 2, Δregion 3, Δregion 4, Δregion5, Δregion 6, Δregion 7, Δregion 8, Δregion 9, Δregion 10, Δregion 11, Δregion 12) | This study |
| KTU-U13 | KT2440 mutant (Δ*upp*, Δregion 1, Δregion 2, Δregion 3, Δregion 4, Δregion 5, Δregion 6, Δregion 7, Δregion 8, Δregion 9, Δregion 10, Δregion 11, Δregion 12, Δregion 13) | This study |
| KTU-U14 | KT2440 mutant (Δ*upp*, Δregion 9) | This study |
| KTU-U15 | KT2440 mutant (Δ*upp*, Δregion 9, Δregion 10) | This study |
| KTU-U16 | KT2440 mutant (Δ*upp*, Δregion 9, Δregion 10, Δregion 11) | This study |
| KTUΔhsdS | KT2440 mutant (Δ*upp*, Δ*hsdS*) | This study |
| KTUΔgcd | KT2440 mutant (Δ*upp*, Δ*gcd*) | This lab |
| KTU-U13Δgcd | KT2440 mutant (Δ*upp*, Δregion 1, Δregion 2, Δregion 3, Δregion 4, Δregion5, Δregion6, Δregion 7, Δregion 8, Δregion 9, Δregion 10, Δregion 11, Δregion 12, Δregion 13, Δ*gcd*) | This study |
| KTU-Lin-TCP | KT2440 mutant (Δ*upp*, Lin^+^, TCP^+^) | This lab |
| KTU-U13-Lin | KT2440 mutant (Δ*upp*, Δregion 1, Δregion 2, Δregion 3, Δregion 4, Δregion 5, Δregion 6, Δregion 7, Δregion 8, Δregion 9, Δregion 10, Δregion 11, Δregion 12, Δregion 13, Lin^+^) | This study |
| KTU-U13-Lin-TCP | KT2440 mutant (Δ*upp*, Δregion 1, Δregion 2, Δregion 3, Δregion 4, Δregion5, Δregion 6, Δregion 7, Δregion 8, Δregion 9, Δregion 10, Δregion 11, Δregion 12, Δregion 13, Lin^+^, TCP^+^) | This study |
| KTUΔhsdS-Lin | KT2440 mutant (Δ*upp*, Δ*hsdS*, Lin^+^) | This study |
| KTUΔhsdS-Lin-TCP | KT2440 mutant (Δ*upp*, Δ*hsdS*, Lin^+^, TCP^+^) | This study |
| KTU-pBBR | KT2440 mutant (Δ*upp*, pBBR^+^) | This study |
| KTU-gfp | KT2440 mutant (Δ*upp*, *gfp*^+^) | This study |
| KTU-U1-gfp | KT2440 mutant (Δ*upp*, Δregion 1, *gfp*^+^) | This study |
| KTU-U3-gfp | KT2440 mutant (Δ*upp*, Δregion 1, Δregion 2, Δregion 3, *gfp*^+^) | This study |
| KTU-U7-gfp | KT2440 mutant (Δ*upp*, Δregion 1, Δregion 2, Δregion 3, Δregion 4, Δregion5, Δregion 6, Δregion 7, *gfp*^+^) | This study |
| KTU-U13-gfp | KT2440 mutant (Δ*upp*, Δregion 1, Δregion 2, Δregion 3, Δregion 4, Δregion 5, Δregion 6, Δregion 7, Δregion 8, Δregion 9, Δregion 10, Δregion 11, Δregion 12, Δregion 13, *gfp*^+^) | This study |
| KTU-Z02 | KT2440 mutant (Δ*upp*, Z02^+^) | This study |
| KTU-pSEVA434 | KT2440 mutant (Δ*upp*, pSEVA434^+^) | This study |
| KTU-U13-Z02 | KT2440 mutant (Δ*upp*, Δregion 1, Δregion 2, Δregion 3, Δregion 4, Δregion 5, Δregion 6, Δregion 7, Δregion 8, Δregion 9, Δregion 10, Δregion 11, Δregion 12, Δregion 13, Z02^+^) | This study |
| KTU-U13-pSEVA434 | KT2440 mutant (Δ*upp*, Δunit 1, Δunit 2, Δunit 3, Δunit 4, Δunit 5, Δunit 6, Δunit 7, Δunit 8, Δunit 9, Δunit 10, Δunit 11, Δunit 12, Δunit 13, pSEVA434^+^) | This study |
| KTUΔhsdS-Z02 | KT2440 mutant (Δ*upp*, Δ*hsdS*, Z02^+^) | This study |
| KTUΔhsdS-pSEVA434 | KT2440 mutant (Δ*upp*, Δ*hsdS*, pSEVA434^+^) | This study |
| Plasmids |  |  |
| pK18mobsacB | Kan^r^, suicide plasmid for gene knockout | This lab |
| pKU | Kan^r^, pK18mobsacB derivative containing *upp* gene | This lab |
| pKU-U1 | Kan^r^, pK18mobsacB derivative containing *upp* gene and deletion fragment of region 1 | This study |
| pKU-U2 | Kan^r^, pK18mobsacB derivative containing *upp* gene and deletion fragment of region 2 | This study |
| pKU-U3 | Kan^r^, pK18mobsacB derivative containing *upp* gene and deletion fragment of region 3 | This study |
| pKU-U4 | Kan^r^, pK18mobsacB derivative containing *upp* gene and deletion fragment of region 4 | This study |
| pKU-U5 | Kan^r^, pK18mobsacB derivative containing *upp* gene and deletion fragment of region 5 | This study |
| pKU-U6 | Kan^r^, pK18mobsacB derivative containing *upp* gene and deletion fragment of region 6 | This study |
| pKU-U7 | Kan^r^, pK18mobsacB derivative containing *upp* gene and deletion fragment of region 7 | This study |
| pKU-U8 | Kan^r^, pK18mobsacB derivative containing *upp* gene and deletion fragment of region 8 | This study |
| pKU-U9 | Kan^r^, pK18mobsacB derivative containing *upp* gene and deletion  fragment of region 9 | This study |
| pKU-U10 | Kan^r^, pK18mobsacB derivative containing *upp* gene and deletion fragment of region 10 | This study |
| pKU-U11 | Kan^r^, pK18mobsacB derivative containing *upp* gene and deletion fragment of region 11 | This study |
| pKU-U12 | Kan^r^, pK18mobsacB derivative containing *upp* gene and deletion fragment of region 12 | This study |
| pKU-U13 | Kan^r^, pK18mobsacB derivative containing *upp* gene and deletion fragment of region 13 | This study |
| pKU-hsdS | Kan^r^, pK18mobsacB derivative containing *upp* gene and deletion fragment of *hsdS* | This study |
| pKU-gcd | Kan^r^, pK18mobsacB derivative containing *upp* gene and deletion fragment of *gcd* | This lab |
| pKU-Lin | Kan^r^, pK18mobsacB derivative containing *upp* gene and the related genes of γ-HCH biodegradation pathway | This study |
| pKU-TCP | Kan^r^, pK18mobsacB derivative containing *upp* gene and the related genes of TCP biodegradation pathway | This study |
| pBBR1MCS-2 | Expression plasmid, Km^R^ | This lab |
| pBBR-gfp | pBBR1MCS-2 derivative containing *gfp* gene | This lab |
| pSEVA434 | Expression plasmid, Sm^r^ | This lab |
| pSEVA434-Z02 | Sm^r^, pSEVA434 derivative containing *idi, dxs, ispA, crtE, crtI, crtB, crtY* and *crtZ* genes | This study |

**Table S9** Primers used in this study

| Primer name | Primer sequence (5’-3’) | Source or reference |
| --- | --- | --- |
| U1-uf | CCGGGTACCGAGCTCGAATTCCCACTGTCACTCGCTCTTTG | This work |
| U1-ur | TCGCTCGCAGCAAACAAGCAGGTCATCACCCAAGAAC | This work |
| U1-df | GGTGATGACCTGCTTGTTTGCTGCGAGCGACATGAAC | This work |
| U1-dr | AAAACGACGGCCAGTGAATTCGCCTGGGTATCGAGCACTTC | This work |
| U1-f | GGTGCGCAGTCATGCCAAAG | This work |
| U1-r | GTGTTCGATGTGCCCAGCGT | This work |
| U2-uf | CCGGGTACCGAGCTCGAATTCCATGGCCGCTACCGATGATG | This work |
| U2-ur | GCGAATCCAACCTGACTTGACTGTGCTCAACGCGC | This work |
| U2-df | TTGAGCACAGTCAAGTCAGGTTGGATTCGCAACAG | This work |
| U2-dr | AAAACGACGGCCAGTGAATTCCTTGGCGAGAACTTCCTGGC | This work |
| U2-f | ACACCAGTTGGCCGTAGTCC | This work |
| U2-r | CAATGATGTGGCCGTGGTGC | This work |
| U3-uf | CCGGGTACCGAGCTCGAATTCATTGTCGTGAAGCCGTCACC | This work |
| U3-ur | GCTTGCAATGGGCACGTGTCGTGCTGTGATCGGCA | This work |
| U3-df | ATCACAGCACGACAC GTGCCCATTGCAAGCACTCC | This work |
| U3-dr | AAAACGACGGCCAGTGAATTCCCACCATGAATGATCGCGCT | This work |
| U3-f | GGACGTGCTGGAACAGCTTG | This work |
| U3-r | CACCAGTTCATTGGCAGGCG | This work |
| U4-uf | CCGGGTACCGAGCTCGAATTCTCAACCGCAGTACGTGCAAC | This work |
| U4-ur | GTTTTCAGGGCCTGGGCAACTCCCTGCCGTAGTTT | This work |
| U4-df | ACGGCAGGGAGTTGC CCAGGCCCTGAAAACCATGC | This work |
| U4-dr | AAAACGACGGCCAGTGAATTCCACGATTGGCCCTTGAGCTT | This work |
| U4-f | TGGCAAAGCGGTACGAACTC | This work |
| U4-r | GAAGAAGCCTGGACTGACCT | This work |
| U5-uf | CCGGGTACCGAGCTCGAATTCAGCCTTGCAGCGAACACTTG | This work |
| U5-ur | TCCACGAGAATCCGCCGAAACGCTTCTAGGTGACG | This work |
| U5-df | CCTAGAAGCGTTTCGGCGGATTCTCGTGGAGAACC | This work |
| U5-dr | AAAACGACGGCCAGTGAATTCGAATGGCTCGCGGGCTTATC | This work |
| U5-f | CATCGTGCTCAGTGACGATG | This work |
| U5-r | GGTTCCGGGAAAGATTCCCT | This work |
| U6-uf | CCGGGTACCGAGCTCGAATTCTCTGCGTGTTCAGAGCCATC | This work |
| U6-ur | TCAACCAGACATTGACGCCCCCAGAAAATTGGCAT | This work |
| U6-df | AATTTTCTGGGGGCGTCAATGTCTGGTTGAGCCCG | This work |
| U6-dr | AAAACGACGGCCAGTGAATTCGAACATCTCGGAGGTGGACG | This work |
| U6-f | ATCGGGCTGAACATTCAGGC | This work |
| U6-r | GAGCAGGCCCAGCTTGATTT | This work |
| U7-uf | CCGGGTACCGAGCTCGAATTCCTCGGCAATGGTCCACATCG | This work |
| U7-ur | TTGCGTAGCGAACGCCGAACCATGGGAGGCACCTA | This work |
| U7-df | GCCTCCCATGGTTCGGCGTTCGCTACGCAATTGAG | This work |
| U7-dr | AAAACGACGGCCAGTGAATTCGATGCTTGCCGCATGAAACG | This work |
| U7-f | TCTTTCCAACCGGCGTAGTC | This work |
| U7-r | CCATGATGCGCGATTTCCTG | This work |
| U8-uf | CCGGGTACCGAGCTCGAATTCGGTTTGCGTGGAGCAGTACC | This work |
| U8-ur | ATCTCGGGTGCGCTCGTAGTGGTGAGTCTGGAGGG | This work |
| U8-df | CAGACTCACCACTACGAGCGCACCCGAGATCTTTG | This work |
| U8-dr | AAAACGACGGCCAGTGAATTCGCTTAAACATTTCGGCGGCC | This work |
| U8-f | GACACAACCGCTACGGCTAC | This work |
| U8-r | CAAGGGCCAGCTGAACATGG | This work |
| U9-uf | CCGGGTACCGAGCTCGAATTCCTATGCACGGTCGGCAGAAG | This work |
| U9-ur | AACGTTCGCCACGGTGATAGCCGGGCAAATGACCT | This work |
| U9-df | ATTTGCCCGGCTATCACCGTGGCGAACGTTATCAG | This work |
| U9-dr | AAAACGACGGCCAGTGAATTCGAATCTGGCTGAAGCGGGTC | This work |
| U9-f | CCCGTTCTTCACCACCAAGG | This work |
| U9-r | GACATGGCGTTGAGGCATCT | This work |
| U10-uf | CCGGGTACCGAGCTCGAATTCGGTCGCGTACCAGGTCTTTC | This work |
| U10-ur | AAGGCGCTGCGCTTTCTGCTGCTGACGATCGAAGG | This work |
| U10-df | GATCGTCAGCAGCAGAAAGCGCAGCGCCTTGCAAC | This work |
| U10-dr | AAAACGACGGCCAGTGAATTCCAGAACCGGATCGTCAGGGA | This work |
| U10-f | CCGACGTAACGCGAGTCTTC | This work |
| U10-r | CCACAAGCGCAGGTCAGTTC | This work |
| U11-uf | CCGGGTACCGAGCTCGAATTCTCAAGGCTCTGCAGCACAAC | This work |
| U11-ur | GTCTGAGTTGTGGCCCGTTATGGCTTGGTTCCAGC | This work |
| U11-df | AACCAAGCCATAACGGGCCACAACTCAGACCATTC | This work |
| U11-dr | AAAACGACGGCCAGTGAATTCCTTGATCTTGCCGATCTGCC | This work |
| U11-f | CAAGCCACCGCTTCGACATC | This work |
| U11-r | CTTGGTATTGAGCAGCGGCA | This work |
| U12-uf | CCGGGTACCGAGCTCGAATTCTCTGGCTGCTGTTCCTCAAG | This work |
| U12-ur | TCCATCGACGGCCTCGTTGTCGATGAGTAGCGCCG | This work |
| U12-df | CTACTCATCGACAACGAGGCCGTCGATGGACTTTC | This work |
| U12-dr | AAAACGACGGCCAGTGAATTCCGAACTGGATGGCAAGCGTA | This work |
| U12-f | ACTGGAAGACGCGCTGTACG | This work |
| U12-r | GTGTTCCCGCCTTACTCGAT | This work |
| U13-uf | CCGGGTACCGAGCTCGAATTCCGTATCGTGACCCGTACCAG | This work |
| U13-ur | ATTGTGAACTGGCGGCCTCCGAAGCGATTGCTCTT | This work |
| U13-df | CAATCGCTTCGGAGGCCGCCAGTTCACAATTCAGC | This work |
| U13-dr | AAAACGACGGCCAGTGAATTCCGGATGCATAGCGGAATCGA | This work |
| U13-f | ATGCCGAGGTCATGGACAAG | This work |
| U13-r | CTGGACGTGCTGGATGACAA | This work |
| hsdS-uf | CCGGGTACCGAGCTCGAATTCAACCGCCGATTTGTTCTTGG | This work |
| hsdS-ur | CCTGAGCCATAAGGGGTTGTCGATGAGTAGCGCCG | This work |
| hsdS-df | CTACTCATCGACAACCCCTTATGGCTCAGGTGTAG | This work |
| hsdS-dr | AAAACGACGGCCAGTGAATTCCGTACAATGGGCGCTTAAGT | This work |
| hsdS-f | TCCACCTAAGCAGCCAACTC | This work |
| hsdS-r | CTGGATTGCAAAGCTGCGTT | This work |
| gcd-uf | TGGATCGGGATGACGTAGAC | This work |
| gcd-dr | GACAACTATGTGCAGGCAGG | This work |
| gcd-f | CCCAGCTCTTGCTGGATGAC | This work |
| gcd-r | CATCAGCAACGACCGCATCA | This work |
| Lin-uf | TGAACGTGCAGAACGCCATC | This work |
| Lin-dr | GTCTGCTCATCGGCGAACAG | This work |
| Lin-f | CCAACCTCGAGCTGGTGTTC | This work |
| Lin-r | GAGATCAAGGCACTGCACCA | This work |
| TCP-uf | TCGCTGATGACCTCGGTTTC | This work |
| TCP-dr | CATACAAGTGCCAGCTCGGC | This work |
| TCP-f | TGTTGTTGTTGCTGCCATCG | This work |
| TCP-r | GATGCTGCGCAAGGTATTGG | This work |
| gfp-f | CACTGGAGTTGTCCCAATTC | This work |
| gfp-r | CCATGTGTAATCCCAGCAGC | This work |
| Z02-3f | CCGACATCATAACGGTTCTG | This work |
| Z02-3r | ACCCACAACATCCCGTTCTC | This work |
| Z02-5f | TAAGGACAGCCCGAATGACG | This work |
| Z02-5r | CGAGCGTTCTGAACAAATCC | This work |


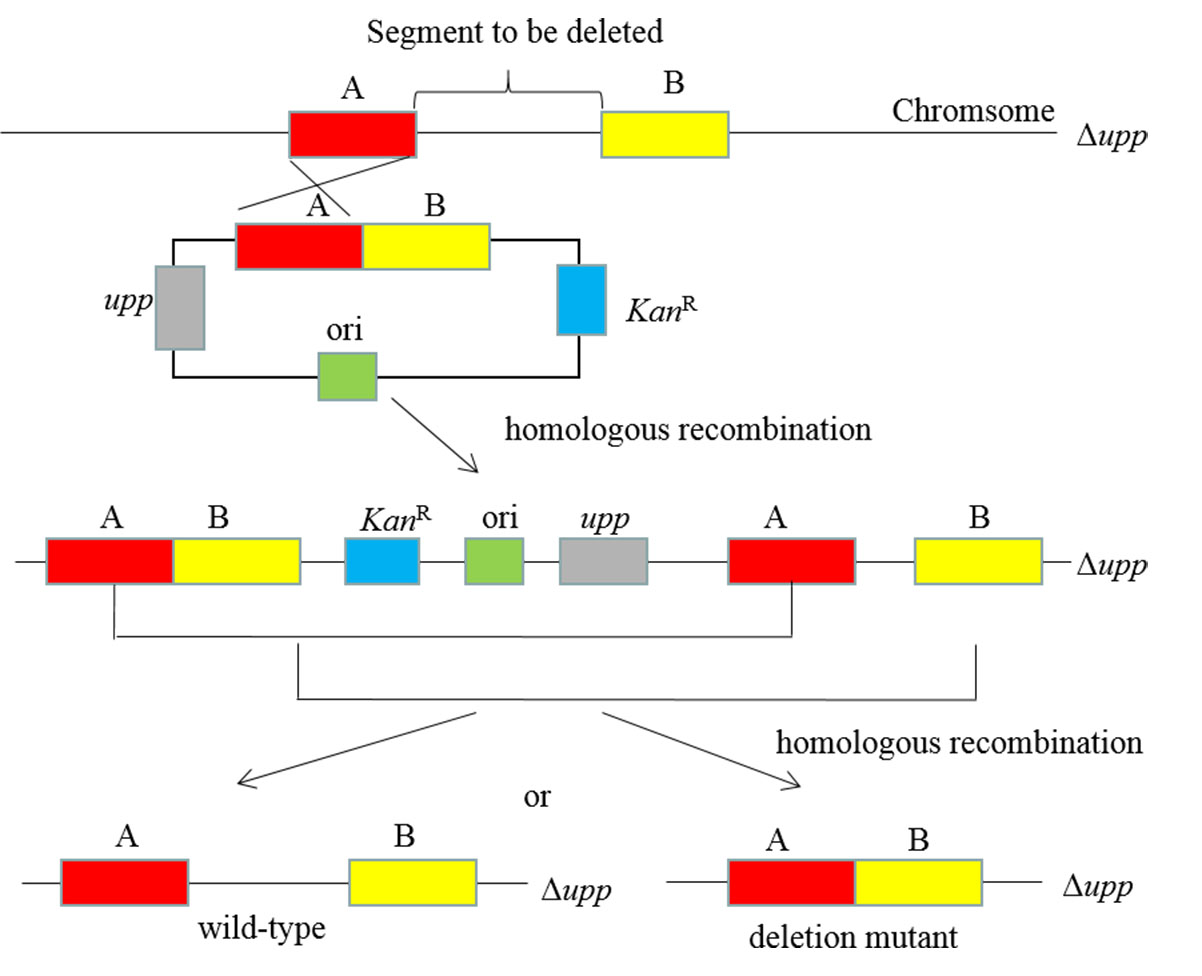


**Fig. S1** Schematic representation of the scarless deletion method for deletion of GIs in *P. putida*.


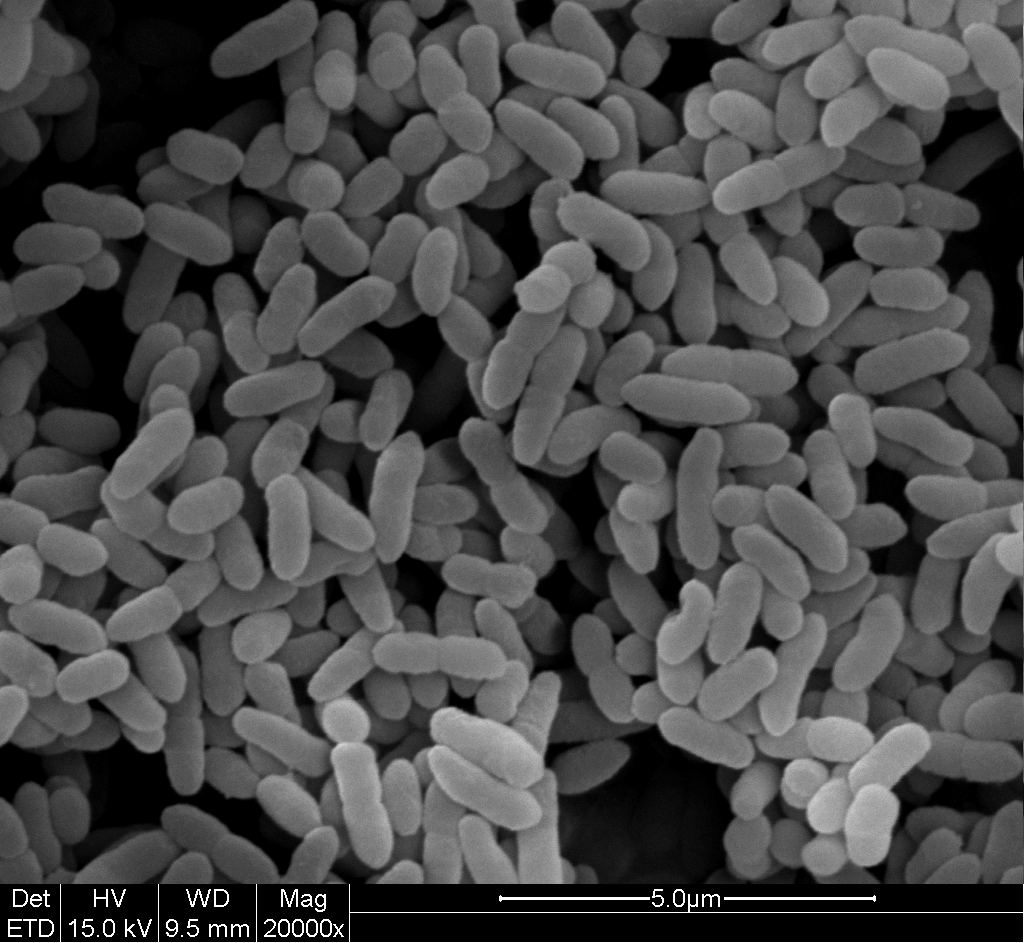

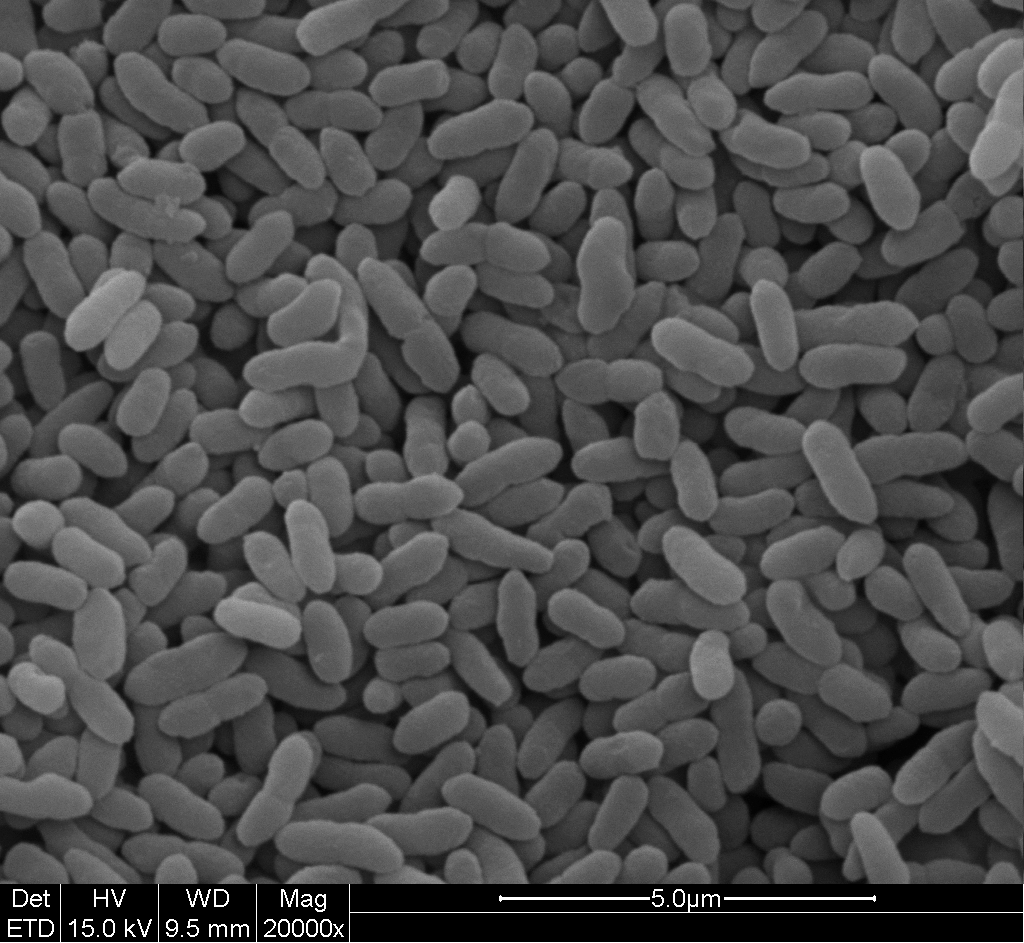


KTU

KTU-U13

**Fig. S2** The cell morphology of the original strain KTU and the GIs-deleted mutant KTU-U13 in LB medium

**
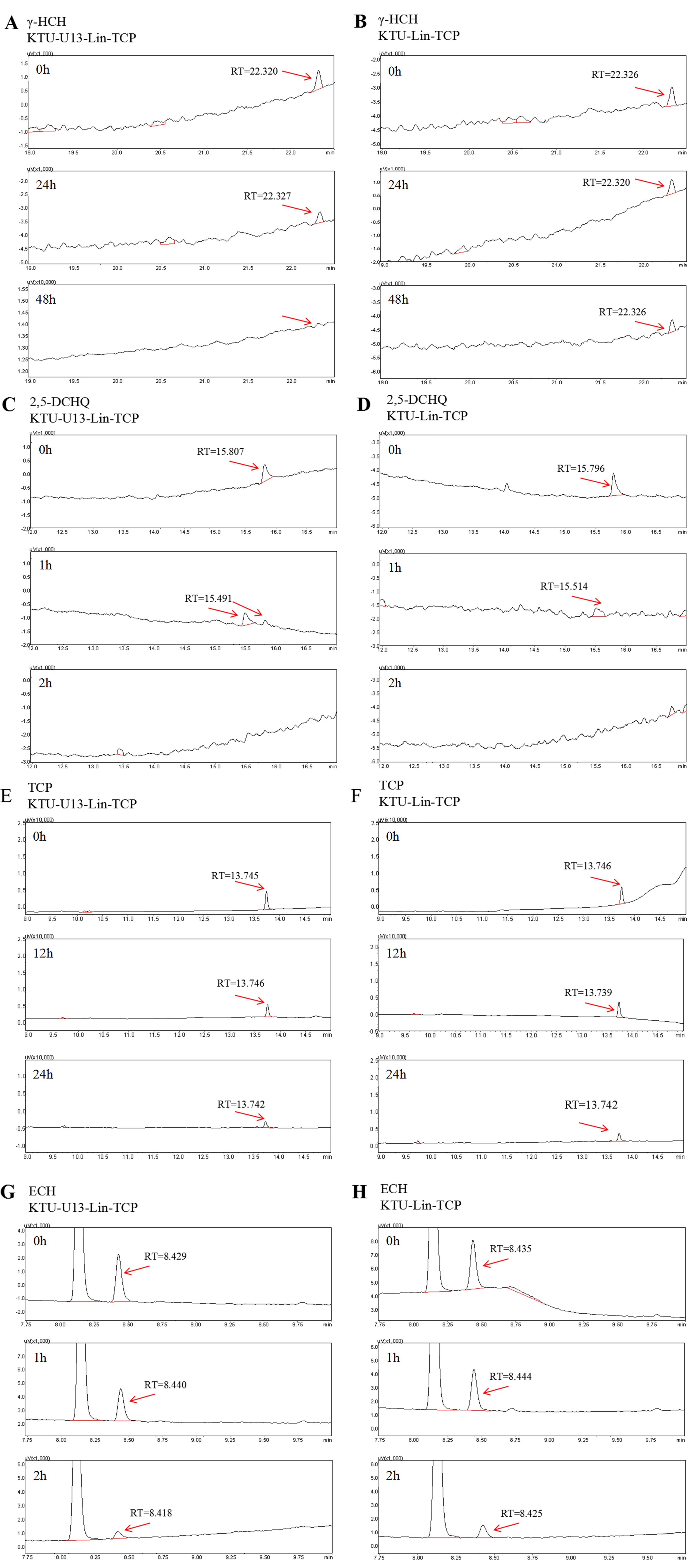
**

**Fig. S3** Degradation of γ-HCH, 2,5-DCHQ, TCP and ECH by the *P. putida* mutants KTU-U13-Lin-TCP and KTU-Lin-TCP. The degradation experiments were performed at 30°C with an initial cell density of OD_600_ = 0.5 in M9G supplemented with 20 mg/l γ-HCH, 50 mg/l 2,5-DCHQ, 0.5 mM TCP, or 0.5 mM ECH.


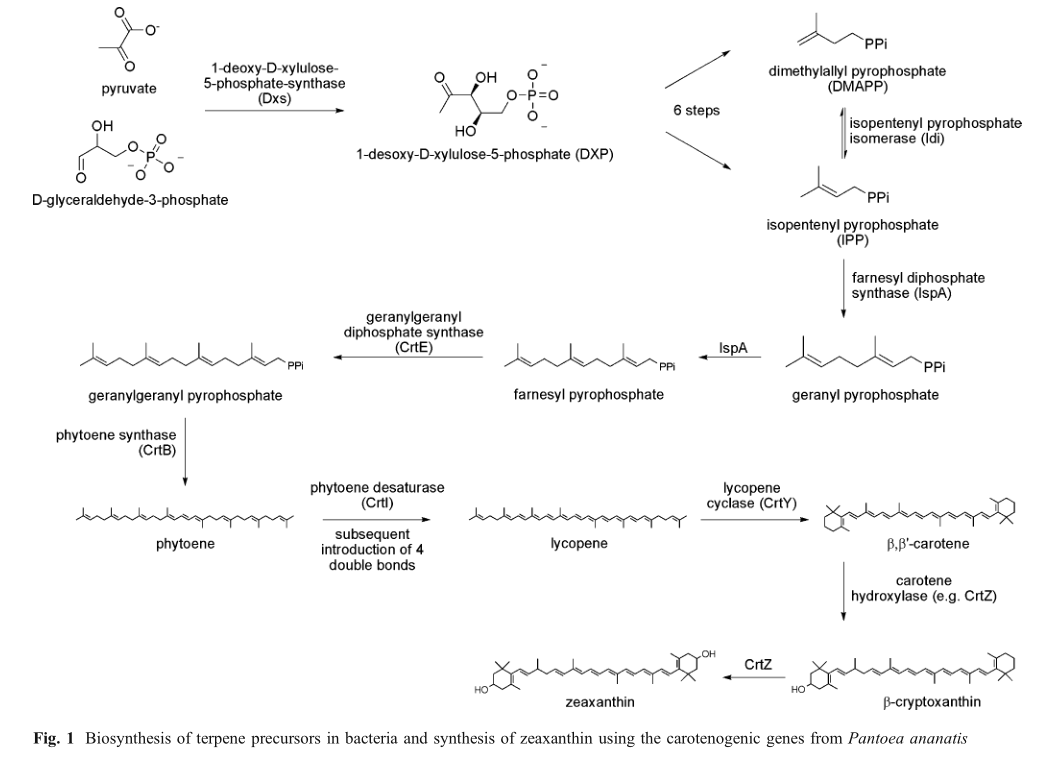


**Fig. S4** Biosynthesis of terpene precursors in bacteria and synthesis of zeaxanthin using the carotenogenic genes from *Pantoea ananatis*. The figure is derived from the following article: Appl Microbiol Biotechnol 2011, 89(4):1137–1147.


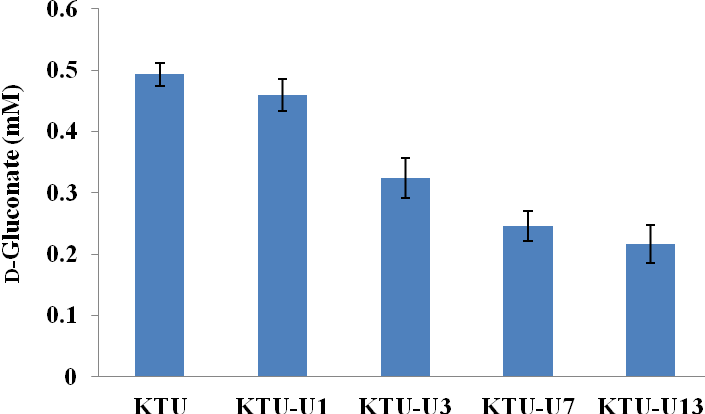


**Fig. S5** Detection for the concentrations of D-Gluconate in the PHA fermentation media of the *P. putida* mutants. D-Gluconate (C_6_H_11_O_7_) was quantified by using a D-Gluconate Assay Kit (Abcam) based on a colorimetric method according to the manufacturer's instructions. The absorbance at 450 nm was measured with a microplate reader. For the biosynthesis of PHA by the strains, the strains were incubated for 60 h at 30°C and 180 rpm in 100 ml of M9 minimal medium supplemented with 20 g/l glucose. After the fermentation was completed, the culture supernatant was obtained by centrifugation and used for D-Gluconate detection. The data are mean values ± standard deviations from three replicates.
